# Nucleolin loss of function leads to aberrant Fibroblast Growth Factor signaling and craniofacial anomalies

**DOI:** 10.1242/dev.200349

**Published:** 2022-06-28

**Authors:** Soma Dash, Paul A. Trainor

**Affiliations:** 1 Stowers Institute for Medical Research, Kansas City, MO 64110, USA; 2 Department of Anatomy and Cell Biology, University of Kansas Medical Center, Kansas City, KS 66160, USA

**Keywords:** Nucleolin, Ribosomal RNA, Neural crest cells, Craniofacial development, FGF signaling, P53, Zebrafish

## Abstract

Ribosomal RNA (rRNA) transcription and ribosome biogenesis are global processes required for growth and proliferation of all cells, yet perturbation of these processes in vertebrates leads to tissue-specific defects termed ribosomopathies. Mutations in rRNA transcription and processing proteins often lead to craniofacial anomalies; however, the cellular and molecular reasons for these defects are poorly understood. Therefore, we examined the function of the most abundant nucleolar phosphoprotein, Nucleolin (Ncl), in vertebrate development. *ncl* mutant (*ncl^−/−^*) zebrafish present with craniofacial anomalies such as mandibulofacial hypoplasia. We observed that *ncl^−/−^* mutants exhibited decreased rRNA synthesis and p53-dependent apoptosis, consistent with a role in ribosome biogenesis. However, we found that Nucleolin also performs functions not associated with ribosome biogenesis. We discovered that the half-life of *fgf8a* mRNA was reduced in *ncl^−/−^* mutants, which perturbed Fgf signaling, resulting in misregulated Sox9a-mediated chondrogenesis and Runx2-mediated osteogenesis. Consistent with this model, exogenous FGF8 treatment significantly rescued the cranioskeletal phenotype in *ncl^−/−^* zebrafish, suggesting that Nucleolin regulates osteochondroprogenitor differentiation. Our work has therefore uncovered tissue-specific functions for Nucleolin in rRNA transcription and post-transcriptional regulation of growth factor signaling during embryonic craniofacial development.

## Abstract (precis)

**Summary:** Loss of function of the ribosomal RNA processing protein Nucleolin results in cartilage and bone hypoplasia in the craniofacial region of zebrafish, which can be rescued by FGF8 supplementation.

## INTRODUCTION

The craniofacial complex consists of the primary sense organs, central and peripheral nervous systems, and musculoskeletal components of the head and neck. Craniofacial development is therefore an intricate process, which involves the coordinated interaction of all three germ layers, but it is also sensitive to environmental and genetic insults, which often result in craniofacial disorders. Despite advances in genomic sequencing, many affected individuals have an unknown genetic diagnosis. Therefore, it is necessary to identify novel genetic factors and better understand the cellular and molecular mechanisms that regulate normal craniofacial development, which may also aid in identifying potential therapeutic targets to prevent or ameliorate craniofacial diseases.

Ribosome biogenesis is essential for cell growth and survival because ribosome quantity and quality dictate the translation of mRNA into proteins. Transcription of ribosomal DNA (rDNA) by RNA Polymerase (Pol) I in the nucleolus generates a 47S pre-ribosomal RNA (pre-rRNA). The 47S pre-rRNA is then cleaved and processed into 18S, 5.8S and 28S rRNAs. These rRNAs, together with Pol III-transcribed 5S rRNA, associate with ribosomal proteins and accessory proteins to form ribosomes (Laferté et al., 2006). The transcription and processing of pre-rRNA requires RNA Pol I together with associated proteins such as UBTF and SL-1 (Friedrich et al., 2005; Panov et al., 2006), as well as rRNA-processing proteins including Treacle (encoded by *Tcof1*) (Jones et al., 2008; Valdez et al., 2004), Nol11 (Freed et al., 2012), Wrd43 (Zhao et al., 2014) and Fibrillarin (Tollervey et al., 1991). Interestingly, when Pol I subunits or associated factors are disrupted or mutated in zebrafish, frogs or mice, it results in developmental defects that mostly affect craniofacial cartilage and bone differentiation (Achilleos and Trainor, 2015; Bouffard et al., 2018; Dixon et al., 2006; Griffin et al., 2015; Watt and Trainor, 2014; Watt et al., 2016, 2018; Yelick and Trainor, 2015; Zhao et al., 2014). This raises the question of why disruptions in these ubiquitously expressed genes, which are required in a global process, result in tissue-specific craniofacial anomalies. One hypothesis is that neural crest cells (NCCs), which are the progenitors of most of the craniofacial bone and cartilage, are more proliferative or metabolically active than non-NCCs (Falcon et al., 2022). In addition, NCCs form via an epithelial-to-mesenchymal transition, which involves major cytoskeletal changes, thus requiring high levels of new protein synthesis and, accordingly, more rRNA transcription. Another hypothesis is that the RNA Pol I subunit, associated proteins and rRNA-processing proteins have other non-ribosomal functions, which, together with the regulation of rRNA synthesis, make the craniofacial skeleton more susceptible to disruption (Sakai and Trainor, 2016; Sakai et al., 2016; Warner and McIntosh, 2009).

Nucleolin is a major nucleolar protein, rRNA-processing protein and nucleotide-binding protein (Ghisolfi-Nieto et al., 1996; Ginisty et al., 1998). Among the top ten highly enriched genes in NCCs (Pijuan-Sala et al., 2019), *Ncl* is the only one involved in rRNA transcription. Therefore, in this study we explored the hypothesis that Nucleolin regulates rRNA transcription, NCC development and craniofacial-specific gene expression and function during embryogenesis. We discovered that Nucleolin is essential for zebrafish embryo survival and is required for NCC-derived craniofacial bone and cartilage development. Furthermore, Nucleolin regulates rRNA transcriptionally, *fgf8a* mRNA post-transcriptionally and the p53 protein post-translationally. Consistent with this model, we demonstrate that exogenous human recombinant FGF8 can ameliorate the cranioskeletal defects as well as recover rRNA transcription in *ncl^−/−^* mutant embryos. Our work, therefore, has uncovered previously unreported tissue-specific functions for Nucleolin in regulating rRNA transcription and FGF signaling in osteochondroprogenitor differentiation during vertebrate embryogenesis, each of which is crucial for craniofacial development.

## RESULTS

### Nucleolin is dynamically expressed during craniofacial development

**Fig. 1.**
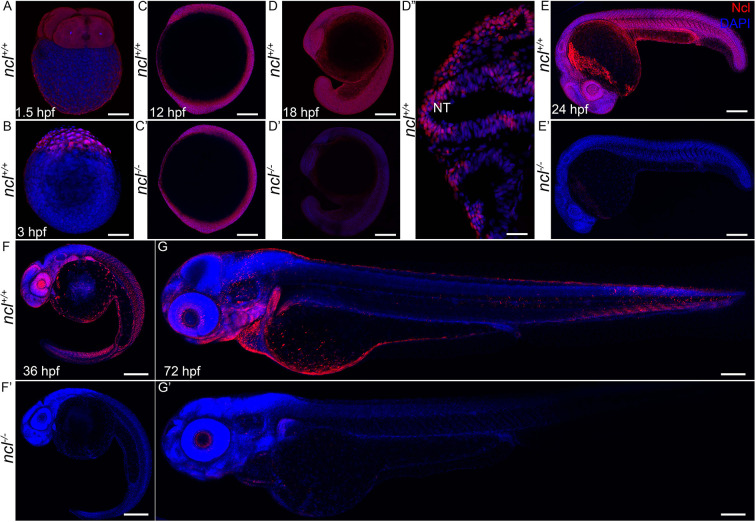
**Ncl expression during zebrafish development.** (A) During embryogenesis, Nucleolin (Ncl, red) was ubiquitously expressed in the cytoplasm of four-cell stage wild-type embryo at 1.5 hpf as observed by immunostaining. (B) Similarly, 3 hpf embryos also had ubiquitous cytoplasmic expression of Nucleolin. (C,C′) At 12 hpf, *ncl^+/+^* and *ncl^−/−^* embryos exhibited similar Nucleolin expression in the nucleus and cytoplasm in most cells of the embryos. (D-D″) At 18 hpf, the expression of Nucleolin in *ncl^+/+^* embryos was ubiquitous and was confined to the nucleus (D″). In the *ncl^−/−^* embryos, the expression pattern of Nucleolin was similar to that of wild-type embryos; however, the expression levels were significantly lower than that of the wild type. (E,E′) By 24 hpf, the expression of Nucleolin was still ubiquitous, with higher levels in the eye and the midbrain-hindbrain boundary in *ncl^+/+^* embryos, whereas it was absent in *ncl^−/−^* embryos. (F) At 36 hpf, the expression of Nucleolin became specific to the craniofacial region in the pharyngeal arches as well as the eye. (G) In 72 hpf (3 dpf) wild-type zebrafish, Nucleolin was highly expressed in the jaw of the embryo. (F′,G′) In the *ncl^−/−^* mutants, there was no expression of Nucleolin. *n*=15 for each panel. The experiment was performed three times. NT, neural tube. Scale bars: 35 µm (A,B); 70 µm (C,C′); 140 µm (D,D′); 50 µm (D″); 250 µm (E,E′); 300 µm (F,F′,G,G′).

To understand the function of Nucleolin in vertebrate development, we characterized its protein expression during zebrafish embryogenesis (Fig. 1). Nucleolin was maternally expressed at 1.5 h post fertilization (hpf) and remained ubiquitously expressed through blastulation (3 hpf), early neurulation (12 hpf) and axial segmentation (18 hpf) (Fig. 1A-D). While at early stages, Nucleolin was present in the cytoplasm, and by 18 hpf, its expression became nuclear (Fig. 1D″). At 24 hpf, the expression of Nucleolin was still ubiquitous; however, it was enriched in the eye and the midbrain-hindbrain boundary (MHB) (Fig. 1E). In 36 and 72 hpf zebrafish embryos, elevated expression of Nucleolin was observed within the eye, pharyngeal arches and the brain (Fig. 1F,G). These expression analyses demonstrated that Nucleolin is dynamically expressed during embryogenesis with enriched expression in craniofacial tissues. Further, this suggests that Nucleolin may be required for proper craniofacial morphogenesis.

### Mutations in zebrafish *ncl* result in craniofacial anomalies

To test our hypothesis that Nucleolin is functionally required for proper craniofacial development, we characterized the phenotype of the *ncl* zebrafish line *ncl^hi2078Tg^* that was generated by insertional mutagenesis of exon 1, which disrupts *ncl* transcription (Amsterdam et al., 2004). *ncl^hi2078T/hi2078Tg^* zebrafish exhibited dramatically reduced levels of the Nucleolin protein between 18 hpf and 24 hpf (Fig. 1C′-G′), thereby establishing them as *ncl^−/−^* mutants.

**Fig. 2.**
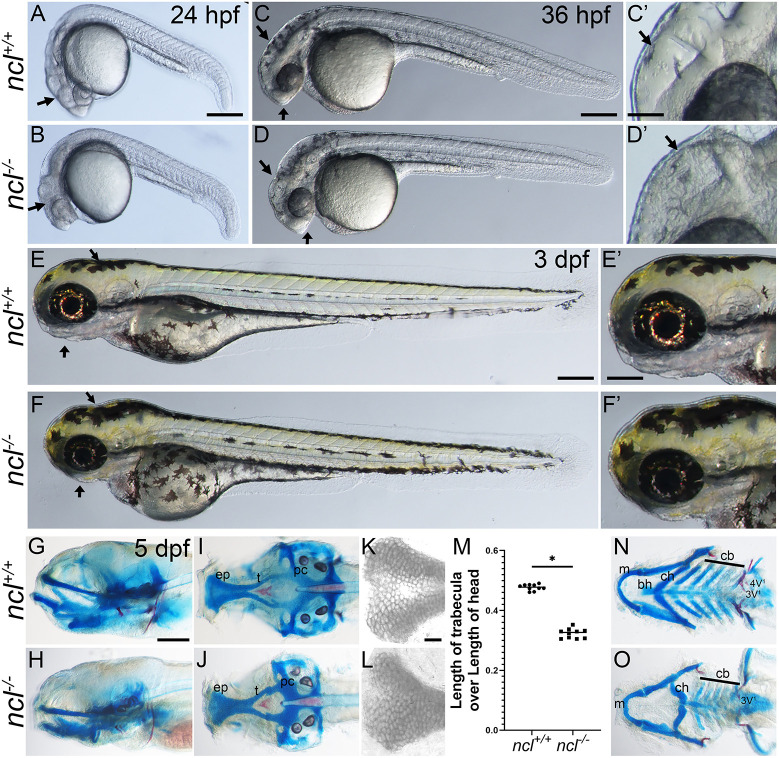
***ncl^−/−^* mutants exhibit craniofacial defects.** (A,B) Compared with 24 hpf *ncl^+/+^* clutch mates, *ncl^−/−^* mutants had necrotic tissue (indicated by black arrows) in the craniofacial region (*n*=25). (C,D) By 36 hpf, the frontonasal prominence and midbrain-hindbrain boundary were misshapen in *ncl^−/−^* mutants compared with their *ncl^+/+^* siblings (indicated by black arrows) (*n*=50). The craniofacial region is magnified in C′,D′. (E,F) At 3 dpf, the *ncl^−/−^* mutants had smaller jaws and a misshapen head (indicated by black arrows) (*n*=15). The craniofacial region is magnified in E′,F′. (G,H) Skeletal preparations of 5 dpf wild-type and *ncl^−/−^* mutant zebrafish reveal defects in the cartilages of the jaw (*n*=50). (I,J) In the neurocranium, the chondrocytes in the ethmoid plate were delayed in development, and the trabeculae were smaller and wider compared with the wild-type zebrafish at the same stage. (K,L) Magnified images of the ethmoid plate showing differential Alcian Blue staining, as well as the loss of medial cells in *ncl^−/−^* larvae. (M) Quantification of the length of trabecula in *ncl^+/+^* and *ncl^−/−^* embryos as a ratio of the length of the head measured from the anterior-most point of the ethmoid plate to the posterior-most point of the parachordal (pc) (*n*=10). Horizontal lines represent the mean. *P<0.05 (two-tailed, paired Student's *t*-test). (N,O) In the viscerocranium, Meckel's cartilage was bent, the basihyal was missing, the polarity of the ceratohyal was inverted and the ceratobranchials were hypoplastic. In addition, the mutants had hypoplastic teeth and the 4V^1^ teeth were missing. The experiment was performed three times. ep, ethmoid plate; t, trabecula; pc, parachordal; m, Meckel's cartilage; bh, basihyal; ch, ceratohyal; cb, ceratobranchial. Scale bars: 200 µm (A-F); 50 µm (C′-F′); 70 µm (G-J,N,O); 25 µm (K,L).

*ncl^−/−^* embryos were phenotypically distinguishable from their wild-type (WT) siblings at 24 hpf by their smaller heads and the presence of necrosis in the craniofacial region (Fig. 2A,B). At 36 hpf, *ncl^−/−^* embryos had a misshapen frontonasal prominence and MHB (Fig. 2C-D′). By 3 days post fertilization (dpf), *ncl^−/−^* mutants displayed craniofacial anomalies, such as mandibular hypoplasia, in addition to a misshapen MHB (Fig. 2E-F′). At 5 dpf, *ncl^−/−^* embryos continued to exhibit craniofacial anomalies and failed to inflate their swim bladders (Fig. S1A,A′), which collectively led to their lethality between 6 and 10 dpf (Fig. S1D,D′).

To further characterize the craniofacial defects in *ncl^−/−^* embryos, we performed skeletal staining with Alcian Blue and Alizarin Red for cartilage and bone, respectively. At 3 dpf, *ncl^−/−^* embryos displayed hypoplastic Meckel's and ceratohyal cartilages compared with their *ncl^+/+^* siblings (Fig. S1B,B′). In 5 dpf *ncl^−/−^* embryos, the craniofacial cartilages were severely hypoplastic (Fig. 2G-M). In the neurocranium, the trabecula and the parasphenoid, which forms the base of the skull (Cubbage and Mabee, 1996), were smaller in *ncl^−/−^* mutants compared with their *ncl^+/+^* siblings (Fig. 2I,J). In *ncl^+/+^* larvae, both edge and medial chondrocyte populations were present and were demarcated by differential Alcian Blue staining. However, in *ncl^−/−^* larvae, cells in the ethmoid plate exhibited uniform Alcian Blue staining, suggesting that the differentiation of medial chondrocytes in the ethmoid plate may be perturbed (Fig. 2K,L). In addition, the trabecula and the parasphenoid were smaller in *ncl^−/−^* larvae compared with *ncl^+/+^* larvae (Fig. 2M), whereas the length of the ethmoid plate was comparable between *ncl^+/+^* and *ncl^−/−^* larvae. In the viscerocranium, the Meckel's cartilage was misshapen and the ceratohyal exhibited reverse polarity, whereas the basihyal was missing in *ncl^−/−^* larvae. Furthermore, the posterior pharyngeal arch-derived ceratobranchials were hypoplastic (Fig. 2N,O) and osteogenesis of the teeth was incomplete in *ncl^−/−^* larvae. At 8 dpf and 10 dpf, the polarity of the ceratohyal was similar between *ncl^+/+^* and *ncl^−/−^* larvae, suggesting that *ncl^−/−^* larvae may have delayed cranioskeletal development. However, the Meckel's cartilage remained misshaped and the ceratobranchials remained hypoplastic in 8 dpf *ncl^−/−^* mutant zebrafish (Fig. S1B-C′ and E-F′). This establishes Nucleolin as being essential for proper cranioskeletal development, and *ncl^−/−^* mutant zebrafish as a new model for understanding the etiology and pathogenesis of craniofacial anomalies.

### *ncl^−/−^* zebrafish have diminished rRNA synthesis

Molecularly, Nucleolin binds to and modifies histones on the promoter of rDNA, and thereby regulates rRNA transcription, which is a rate-limiting step of ribosome biogenesis (Cong et al., 2012). In addition, Nucleolin interacts with the U3 small nucleolar RNA (snoRNA), and thus binds to pre-rRNA to process and splice it (Ginisty et al., 1998; Roger et al., 2003; Shao et al., 2020). Therefore, we hypothesized that *ncl* loss of function would lead to diminished rRNA transcription.

**Fig. 3.**
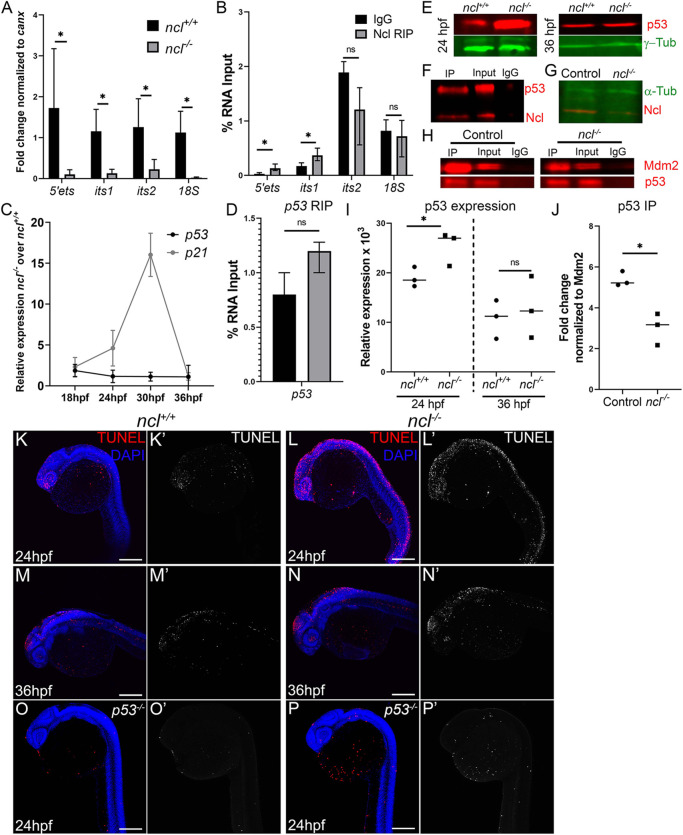
**Nucleolin is required for rRNA transcription and p53 regulation.** (A) qPCR for 5′ETS, ITS1, ITS2 and 18S segment of the pre-rRNA in *ncl^+/+^* and *ncl^−/−^* zebrafish (*n*=10 per sample) indicates that rRNA transcripts were significantly lower in *ncl^−/−^* embryos compared with their *ncl^+/+^* siblings. *canx* was used as an internal control. (B) RNA immunoprecipitation (RIP) using a Nucleolin-specific antibody indicates that Nucleolin binds to the 5′ETS and ITS1 region of the 47S rRNA but not to the ITS2 or 18S in wild-type zebrafish (*n*=100 per replicate and condition). The *y*-axis indicates fold change of RNA pulldown compared with its absolute expression in the embryos. (C) *p53* transcript expression was not significantly altered in *ncl^−/−^* mutant zebrafish between 18 and 36 hpf; however, expression of its downstream target *p21* was significantly higher between 24 and 30 hpf in the *ncl^−/−^* mutants compared with wild-type zebrafish (*n*=5 per sample). (D) Nucleolin and IgG binding to *p53* mRNA was similar in wild-type zebrafish, as observed by RNA immunoprecipitation (*n*=100 per replicate and condition). The *y*-axis indicates fold change of RNA pulldown compared with its absolute expression in the embryos. (E) p53 protein levels were higher in *ncl^−/−^* mutants at 24 hpf compared with their *ncl^+/+^* siblings and comparable between *ncl^+/+^* and *ncl^−/−^* embryos at 36 hpf as observed by western blotting (*n*=5 per sample). γ-tubulin was used as a loading control. (F) Immunoprecipitation (IP) with a Nucleolin-specific antibody followed by western blotting for p53 and Nucleolin indicates that p53 and Nucleolin bind to each other in wild-type zebrafish (*n*=25 per replicate and condition). (G) In *ncl^−/−^* mutants, Nucleolin expression was significantly reduced compared with controls (*n*=25 per replicate). α-tubulin was used as a loading control. (H) At 28 hpf, control zebrafish displayed higher binding of Mdm2 and p53 compared with that seen in mutant zebrafish (*n*=25 per replicate and condition). (I) Quantification of p53 protein levels in 24 hpf and 36 hpf *ncl^+/+^* and *ncl^−/−^* embryos (*n*=3). (J) Quantification of p53-Mdm2 binding in *ncl^+/+^* and *ncl^−/−^* embryos (*n*=3). (K-L′) *ncl^−/−^* mutants have more TUNEL+ cells (red in K,L; white in K′,L′) at 24 hpf compared with their *ncl^+/+^* siblings (*n*=15 per genotype). (M-N′) By 36 hpf, apoptosis (red in M,N; white in M′,N′) is confined to the midbrain-hindbrain boundary in both *ncl^+/+^* and *ncl^−/−^* embryos (*n*=15 per genotype). (O-P′) On a *p53^−/−^* mutant background, the number of TUNEL+ cells (red in O,P; white in O′,P′) in both *ncl^+/+^* and *ncl^−/−^* embryos (*n*=15 per genotype) at 24 hpf is reduced. All experiments were performed three times. Scale bars: 70 µm. Data are represented as mean±s.d. in A-D; circles and squares represent individual data points and horizontal lines represent the mean in I,J. ns, not significant; *P<0.05 (two-tailed, paired Student's *t*-test).

To validate our hypothesis, we quantified rRNA transcription by assaying for its 5′ external transcribed spacer (ETS) by quantitative reverse transcription PCR (qRT-PCR) at four different stages between 18 and 36 hpf, and observed that 5′ETS expression was significantly reduced beginning at 18 hpf, and gradually diminished at later developmental stages (Fig. S2A). We further assayed for the intergenic regions of the 47S pre-rRNA – 5′ETS and the internal transcribed spacer (ITS) 1 and ITS2 – as well as 18S rRNA at 36 hpf, and observed a significant reduction of all four amplicons in *ncl^−/−^* embryos compared with their *ncl^+/+^* siblings, suggesting that Nucleolin is necessary for rRNA transcription in zebrafish (Fig. 3A). We then performed RNA immunoprecipitation using a Nucleolin-specific antibody, and observed that, at 28 hpf, Nucleolin bound to the 5′ETS and ITS1, but not to ITS2 and the 18S regions of the 47S pre-RNA (Fig. 3B), consistent with the known function of Nucleolin *in vitro* (Cong et al., 2012).

Decreased rRNA transcription *in vitro* has previously been shown to result in increased free ribosomal proteins in cells, which then interact with Mdm2, changing its conformation such that Mdm2 can no longer bind to p53 and ubiquitylate it for degradation (Donati et al., 2011). Consequently, p53 accumulates in the cell and results in p53-dependent cell death. To test whether this mechanism holds true *in vivo* in *ncl^−/−^* embryos, we first performed quantitative PCR (qPCR) and western blotting to assess for p53 expression. We observed that *p53* (also known as *tp53*) mRNA transcript levels were not significantly changed in *ncl^−/−^* embryos compared with their *ncl^+/+^* siblings between 18 and 36 hpf (Fig. 3C; Fig. S2B). However, in contrast, p53 protein levels were significantly upregulated in *ncl^−/−^* mutants compared with *ncl^+/+^* controls at 24 hpf, but the difference subsided by 36 hpf (Fig. 3E,I). The downstream target of p53, *p21* (or *cdkn1a*), was initially upregulated between 24 and 30 hpf, but its expression also subsided by 36 hpf (Fig. 3C; Fig. S2C). Nucleolin regulates p53 activity by various mechanisms, including increasing *p53* mRNA and/or p53 protein stability (Saxena et al., 2006; Takagi et al., 2005). We observed that, in wild-type tissues, Nucleolin did not bind to zebrafish *p53* mRNA at 28 hpf (Fig. 3D). Instead, Nucleolin bound to the p53 protein (Fig. 3F).

To test our hypothesis that the Mdm2-p53 protein interaction might be reduced in *ncl^−/−^* embryos as a result of ribosomal stress, we performed immunoprecipitation for Mdm2 in control and *ncl^−/−^* embryos and immunoblotted for both Mdm2 and p53. Prior to examining the binding efficiency of Mdm2 and p53 in control and *ncl^−/−^* embryos, we confirmed that Nucleolin was indeed absent in *ncl^−/−^* embryos at 28 hpf (Fig. 3G). In our immunoprecipitation experiments, we observed decreased pulldown of Mdm2 and p53 in *ncl^−/−^* embryos compared with control embryos, indicating that the initial accumulation of p53 was a result of reduced interaction between Mdm2 and p53 (Fig. 3H-J). The subsidence of p53 activity at later stages might therefore be due to a lack of Nucleolin-dependent stabilization of the p53 protein, which would normally increase its half-life.

### p53-dependent cell death is increased in *ncl^−/−^* mutant embryos

To examine whether increased p53 activation and accumulation resulted in cell death and therefore necrotic craniofacial tissue in *ncl^−/−^* mutants (Fig. 2A), we performed terminal deoxynucleotidyl transferase dUTP nick end labeling (TUNEL) staining at 24 hpf, and observed a general increase in apoptosis in *ncl^−/−^* embryos compared with *ncl^+/+^* controls (Fig. 3K-L′). However, by 36 hpf, TUNEL+ cells were restricted to the MHB in *ncl^−/−^* embryos (Fig. 3M-N′).

p53 activation can lead to increased apoptosis as well as decreased proliferation that collectively result in tissue hypoplasia. Therefore, we performed immunostaining with the G2/M phase marker phospho-histone H3 (pHH3), but observed no significant change in pHH3+ cells in 24 hpf *ncl^−/−^* mutants compared with controls (Fig. S3A,B). Consistent with the subsidence of increased p53 and p21 expression at 36 hpf, the number of pHH3+ cells was significantly increased in *ncl^−/−^* embryos, especially at the MHB, at 48 hpf (Fig. S3C,D,G). We also performed 5-ethynyl-2′-deoxyuridine (EdU) staining on *ncl^+/+^* and *ncl^−/−^* zebrafish at 48 hpf to label cells in the S phase of the cell cycle, and observed that the mutants had a higher number of EdU+ cells (Fig. S3E,F,H). The increase in proliferation may explain how *ncl^−/−^* embryos survived beyond 24 hpf, even though they exhibited elevated apoptosis.

We hypothesized that the increased apoptosis in *ncl^−/−^* embryos might be p53 dependent and that genetically inhibiting p53 would suppress cell death and rescue the craniofacial anomalies in *ncl^−/−^* embryos. Therefore, we crossed the *tp53^M214K/M214K^* allele (hereafter referred to as *p53^−/−^*) into the background of *ncl^−/−^* mutant zebrafish to generate *ncl^−/−^;p53^−/−^* double mutants. Consistent with our hypothesis, TUNEL staining revealed a reduction in the number of apoptotic cells in 24 hpf *ncl^−/−^;p53^−/−^* embryos, compared with *ncl^−/−^* embryos (Fig. 3O-P′). However, EdU labeling indicated that *ncl^−/−^;p53^−/−^* embryos had increased proliferation at 48 hpf, similar to *ncl^−/−^* embryos (Fig. S3I,J), suggesting that the proliferation effects were not p53 dependent.

Removal of both the copies of *p53* altered jaw morphology in *ncl^−/−^* embryos (*n*=82) (Fig. S4). Although the ceratohyal remained smaller compared with control siblings, its polarity was restored to normal. The size of the head and the shape of the Meckel's cartilage improved in *ncl^−/−^;p53^−/−^* embryos compared with *ncl^−/−^;p53^+/+^* larvae. However, chondrogenesis of the ceratobranchials was not improved, and in addition, chondrogenesis was disrupted in the neurocranium. *ncl^−/−^;p53^−/−^* mutant zebrafish failed to inflate their swim bladders, similar to *ncl^−/−^* mutant zebrafish, and died between 10 and 12 dpf. This suggests that, although p53 accumulation resulted in apoptosis during early development, the skeletal differentiation defects in *ncl^−/−^* mutant zebrafish were not p53 dependent.

### NCC development is unaffected in *ncl^−/−^* mutant embryos

In vertebrates, NCCs differentiate into most of the bones and cartilages of the craniofacial skeleton. To determine whether defects in NCC induction and migration underlay the cranioskeletal malformations in *ncl^−/−^* mutants, we labeled NCCs by crossing *sox10:egfp* transgenic zebrafish into *ncl^−/−^* mutant zebrafish. In addition, we immunostained *ncl^+/+^* and *ncl^−/−^* embryos with the antibody Zn-8, which labels the endodermal pouches to demarcate individual pharyngeal arches (Trevarrow et al., 1990; Warga and Nüsslein-Volhard, 1999). *sox10:egfp* labeling of pre- and post-migratory NCCs and volumetric rendering of the pharyngeal arches revealed no significant change in the size of the pharyngeal arches in *ncl^−/−^* mutant embryos compared with *ncl^+/+^* embryos (Fig. S5A,B). Comparisons of the *sox10:egfp* fluorescence intensities in the pharyngeal arches of *ncl^+/+^* embryos with those of the *ncl^−/−^* embryos suggested that NCC induction and migration into the pharyngeal arches were unaffected in *ncl^−/−^* mutant embryos (Fig. S5C). However, the pineal gland as well as the nasopharyngeal regions of *ncl^−/−^* mutants had reduced *sox10:egfp* expression, suggesting that, although NCC migration into the arches was unaffected, migration to other cranial regions may have been altered. We further validated *sox10;gfp* transgene expression data by assessing endogenous Sox10 protein expression in *ncl^+/+^* and *ncl^−/−^* embryos. The number of Sox10+ cells was similar between 36 hpf *ncl^+/+^* and *ncl^−/−^* mutant embryos (Fig. S5D), confirming that NCC induction and migration into the pharyngeal arches occurred normally in *ncl^−/−^* mutants. We observed no alteration in the formation or segregation of the endodermal pouches in 36 hpf *ncl^−/−^* mutants, as evidenced by normal patterns of Zn-8 expression (Fig. S5A). This implied that the osteochondrogenic anomalies in *ncl^−/−^* mutant embryos were not the result of abnormal pharyngeal pouch development.

### Chondrogenic and osteogenic defects in *ncl^−/−^* mutants

**Fig. 4.**
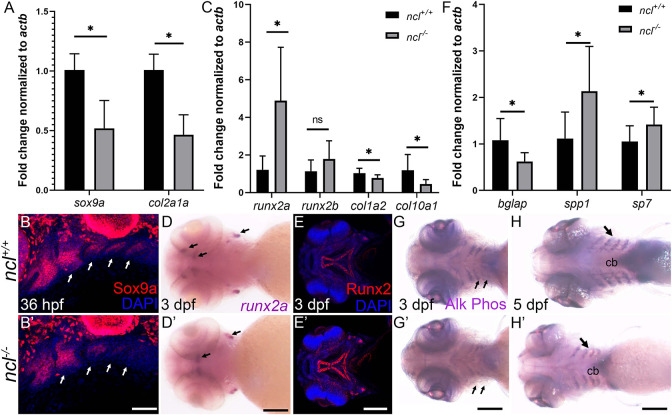
**Chondrogenesis and osteogenesis defects in *ncl^−/−^* embryos.** (A) qPCR revealed a significant downregulation of *sox9a* and *col2a1* chondrogenesis markers in 36 hpf *ncl^−/−^* embryos compared with *ncl^+/+^* embryos (*n*=10 per replicate). *actb* was used as a housekeeping control. (B,B′) Sox9a protein expression was significantly reduced in branchial arches 2-5 (white arrows) in *ncl^−/−^* embryos at 36 hpf (*n*=15). (C) qPCR of osteogenesis markers in 36 hpf *ncl^+/+^* and *ncl^−/−^* embryos indicates significant upregulation in *runx2a* transcripts and downregulation in both *col1a2* and *col10a1* transcripts in *ncl^−/−^* embryos (*n*=10 per replicate). (D,D′) *runx2a* mRNA expression was significantly increased *ncl^−/−^* embryos at 3 dpf (*n*=15). Black arrows indicate expression of *runx2a* in the ceratohyal and otic vesicles. (E,E′) At 3 dpf, Runx2 protein expression was significantly increased in the palatoquadrate and the parasphenoid in *ncl^−/−^* embryos. (F) qPCR indicates a significant upregulation of the early osteoblast markers *bglap* and *spp1* and downregulation of the late osteoblast marker *sp7* in 36 hpf *ncl^−/−^* embryos compared with controls (*n*=10 per replicate). (G-H′) Alkaline phosphatase staining of *ncl^+/+^* and *ncl^−/−^* embryos reveals reduced staining in the lower jaw (ventral view, black arrows) at 3 dpf (G,G′) and 5 dpf (H,H′) (*n*=15). All experiments were performed three times. cb, ceratobranchial. Scale bars: 200 µm (B,B′); 100 µm (D-E′,G,G′); 140 µm (H,H′). Data are represented as mean±s.d. ns, not significant; *P<0.05 (two-tailed, paired Student's *t*-test).

Given the chondrogenic defects observed in *ncl^−/−^* mutant embryos, we next investigated the basis for altered chondrogenesis in *ncl^+/+^* and *ncl^−/−^* embryos. Sox9a is a transcription factor that promotes NCC differentiation into chondrocytes (Yan et al., 2002), and the mRNA levels of *sox9a* and its downstream target *col2a1a* were reduced in *ncl^−/−^* mutant embryos at 36 hpf (Fig. 4A). The overall expression of the Sox9a protein was also reduced in *ncl^−/−^* mutants, but most significantly in pharyngeal arches 2-5, which differentiate into the ceratohyal and ceratobranchial cartilages (Fig. 4B,B′). This provides a molecular explanation for the cartilage hypoplasia in *ncl^−/−^* mutant embryos.

To determine the basis for the osteogenic defects in *ncl^−/−^* mutant embryos, we evaluated the progression of osteogenesis in *ncl^+/+^* and *ncl^−/−^* embryos. Runx2a and Runx2b are transcription factors that govern NCC differentiation to bone (Flores et al., 2006). *runx2a* mRNA expression was upregulated in *ncl^−/−^* mutant embryos (Fig. 4D,D′), as was *runx2b*, but *runx2b* upregulation was not statistically significant at 36 hpf (Fig. 4C). We examined the protein expression of Runx2a and Runx2b in *ncl^−/−^* and *ncl^+/+^* embryos by using a pan-Runx2 antibody that was designed to recognize human RUNX2 in the 300-400 amino acid region, and has 85% similarity with the zebrafish Runx2a and Runx2b proteins. Similar to the results seen for *runx2a* mRNA expression, Runx2 protein levels at 3 dpf were upregulated in the craniofacial region, especially in the palatoquardate and the parasphenoid (Fig. 4E,E′). Runx2 overexpression has previously been observed to result in premature osteoblast differentiation (Liu et al., 2001), which leads to a reduced pool of osteoblasts and, consequently, abnormal skeletal development. In agreement with these findings, the gene expression of the Runx2a and Runx2b downstream targets *col1a2* and *col10a1a* was reduced in *ncl^−/−^* embryos compared with *ncl^+/+^* embryos. Furthermore, we observed an increase in the expression of the early osteoblast markers *spp1* (osteopontin) and *sp7*, and a concomitant decrease in the expression of the late osteoblast marker *bglap* (osteocalcin) in 36 hpf *ncl^−/−^* mutant embryos (Fig. 4F). To confirm the presence of prematurely differentiated osteoblasts, we stained *ncl^+/+^* and *ncl^−/−^* mutant embryos for alkaline phosphatase activity, which is endogenously high in primary osteoblasts and in arteries (Ohlebusch et al., 2020). At 3 dpf and 5 dpf (Fig. 4G-H′), alkaline phosphatase staining was significantly diminished in the ceratobranchials of *ncl^−/−^* embryos compared with *ncl^+/+^* embryos, and overlaid the pattern of Alcian Blue staining in *ncl^−/−^* embryos (Fig. 2O) as well as *fli1a:gfp* expression, which labels NCCs and endothelial cells (Askary et al., 2017). Overall, our data reveal that Nucleolin loss of function results in a reduced pool of osteochondroprogenitors that fail to mature or differentiate properly.

### Fgf8a expression is reduced in *ncl^−/−^* mutant embryos

**Fig. 5.**
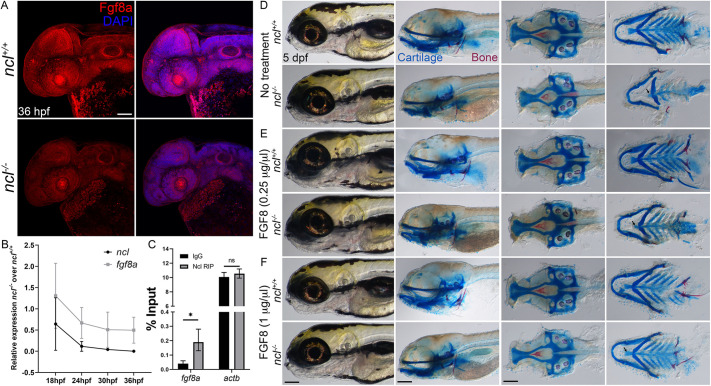
**Nucleolin regulates Fgf8a expression.** (A) Immunostaining of 36 hpf *ncl^+/+^* and *ncl^−/−^* embryos with an Fgf8a-specific antibody reveals reduced expression of Fgf8a in *ncl^−/−^* embryos (*n*=15). (B) qPCR using craniofacial tissues from *ncl^+/+^* and *ncl^−/−^* embryos at 18, 24, 30 and 36 hpf indicates that *ncl* and *fgf8a* expression gradually reduce over time (*n*=10 per replicate). (C) RNA immunoprecipitation followed by qPCR indicates higher binding of *fgf8a* mRNA to Nucleolin compared with the IgG control. *actb* was used as a negative control (*n*=100 per replicate per condition). The *y*-axis indicates fold change of RNA pulldown compared with its absolute expression in the embryos. (D-F) Skeletal preparations of 5 dpf *ncl^+/+^* and *ncl^−/−^* larvae as controls (D) for 0.25 µg/µl (E) and 1 µg/µl (F) FGF8 exogenous treatment. Exogenous FGF8 rescued the cranioskeletal phenotype of *ncl^−/−^* larvae (*n*=45). Black arrows indicate the improvement of the basihyal phenotype in FGF8-treated larvae. All experiments were performed three times. Scale bars: 100 µm (A); 70 µm (D-F). Data are represented as mean±s.d. ns, not significant; *P<0.05 (two-tailed, paired Student's *t*-test).

In mice, Fgf8 signaling upregulates chondrogenic genes such as *Sox9* and *Col2a1* while inhibiting osteogenic genes such as *Runx2* (Xu et al., 2018; Zehentner et al., 1999). Similarly, Fgf signaling regulates *sox9a* in zebrafish (Esain et al., 2010), and interestingly, *fgf8a* mutant zebrafish have craniofacial defects comparable with those seen in *ncl^−/−^* embryos, especially in the cartilages of the viscerocranium (Crump et al., 2004). This suggested a potential link between Nucleolin and Fgf signaling during chondrogenesis, and we therefore examined the expression of Fgf8a in *ncl^+/+^* and *ncl^−/−^* embryos. We observed a significant general downregulation of Fgf8a in *ncl^−/−^* mutant embryos (Fig. 5A), and, in examining the expression of *fgf8a* mRNA by *in situ* hybridization at 36 hpf (Fig. S6) and qPCR at four stages between 18 and 36 hpf in *ncl^−/−^* mutant embryos, we discovered that as the expression of *ncl* decreased in the mutants, the expression of *fgf8a* also declined (Fig. 5B; Fig. S2D,E). This was suggestive of co-regulation of *ncl* and *fgf8a* expression. *In silico* analysis of *fgf8a* mRNA revealed that the *fgf8a* 5′ untranslated region (UTR) contains a Nucleolin consensus binding site – UCCCGA (Ghisolfi-Nieto et al., 1996). We tested for Nucleolin binding to *fgf8a* mRNA through RNA immunoprecipitation and observed that *fgf8a* mRNA was pulled down with Nucleolin, whereas that of the housekeeping gene *actb* was not (Fig. 5C). *actb* is an abundant RNA molecule and is readily available in zebrafish embryos. This suggests that Nucleolin specifically binds to *fgf8a* mRNA and post-transcriptionally regulates *fgf8a* expression.

To determine whether the phenotype of *ncl^−/−^* mutant embryos was a direct consequence of *fgf8a* downregulation, we treated *ncl^+/+^* and *ncl^−/−^* embryos at 18 hpf with human recombinant FGF8, which has 76% identity to the zebrafish Fgf8a protein. Compared with untreated *ncl^−/−^* mutant embryos, we observed a considerable rescue of the craniofacial cartilage phenotype at 60 hpf (*n*=15/15) (Fig. S7A), the degree of which correlated with the concentration of FGF8. The same was true for 5 dpf mutants, with restoration of ceratohyal polarity at 0.25 μg/μl FGF8 (*n*=45/45) (Fig. 5D,E), and rescue of basihyal (*n*=44/45) and ceratobranchial (*n*=45/45) chondrogenesis at 1 μg/μl (Fig. 5F). In addition, the lengths of the trabecula and the parasphenoid were restored in FGF8-treated *ncl^−/−^* larvae (*n*=45/45). However, although osteogenesis of the tooth improved, the mutant embryos were still missing their 4V^1^ teeth (*n*=45/45), indicating that FGF8 partially rescued the *ncl^−/−^* tooth phenotype. At 8 dpf, the phenotypes seen for the craniofacial cartilages (Fig. S7C) as well as the putative posterior swim bladder (Fig. S7B) were rescued in FGF8-treated *ncl^−/−^* larvae. The FGF8-treated *ncl^−/−^* fry survived at least until 15 dpf, which was 5 days longer than untreated *ncl^−/−^* fry, and all the craniofacial skeleton elements were rescued (Fig. S7D,E). However, these fry were smaller than *ncl^+/+^* fry, and their anterior swim bladders failed to inflate. Nonetheless, our data indicate that exogenous FGF8 was sufficient to rescue the craniofacial skeleton defects and increase the lifespan of *ncl^−/−^* mutants.

### FGF8 treatment restores rRNA synthesis in *ncl^−/−^* mutant embryos

*Fgf8* is expressed in the endoderm-derived epithelium of the pharyngeal arches and oral ectoderm, and Fgf receptors are expressed in the pharyngeal endoderm as well as in osteochondroprogenitors (Eberhart et al., 2006; Hall et al., 2006; Larbuisson et al., 2013; Stock et al., 2006; Walshe and Mason, 2003). In addition, Fgf8 has been shown to have mitogenic potential in mice (Cruz-Martinez et al., 2014; Mahmood et al., 1995; Storm et al., 2006). Therefore, FGF8 rescue of cranioskeletal defects in *ncl^−/−^* embryos could be a result of: (1) Fgf signaling through FGF8 interactions with Fgf receptors on osteochondroprogenitors; (2) FGF8-mediated rescue of rRNA transcription; (3) increased proliferation in *ncl^−/−^* mutants as a result of FGF8 treatment; or (4) FGF8-mediated regulation of other mesenchymal signaling pathways important for osteochondroprogenitor differentiation.

**Fig. 6.**
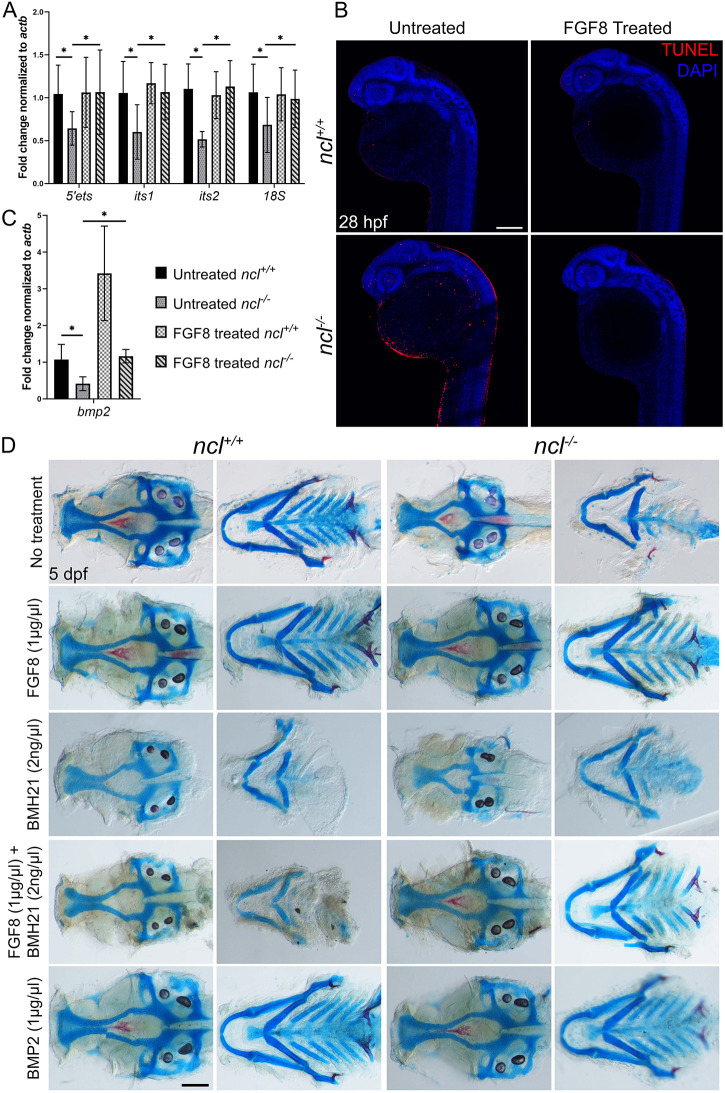
**FGF8 rescues rRNA transcription in *ncl^−/−^* embryos.** (A) qPCR for 5′ETS, ITS1, ITS2 and 18S in untreated and FGF8-treated *ncl^+/+^* and *ncl^−/−^* zebrafish (*n*=10 per sample) indicates rescue of pre-RNA transcription in FGF8-treated *ncl^−/−^* zebrafish at 36 hpf. (B) TUNEL staining of untreated and FGF8-treated *ncl^+/+^* and *ncl^−/−^* zebrafish at 28 hpf indicates reduced TUNEL+ cells in FGF8-treated *ncl^−/−^* embryos compared with untreated *ncl^−/−^* embryos (*n*=15). (C) qPCR for *bmp2* in 36 hpf *ncl^+/+^* and *ncl^−/−^* embryos (*n*=10 per sample) that were untreated or treated with 1 µg/µl FGF8 indicates significant downregulation in *bmp2* in untreated *ncl^−/−^* embryos and significant upregulation in FGF8-treated *ncl^+/+^* embryos. In FGF8-treated *ncl^−/−^* embryos, the *bmp2* transcript levels were rescued and comparable with untreated *ncl^+/+^* embryos. *actb* was used as a housekeeping control. (D) Skeletal preparations of 5 dpf *ncl^+/+^* and *ncl^−/−^* larvae that were untreated or treated with FGF8, BMH21, FGF8+BMH21 or BMP2. Exogenous FGF8 and BMP2 treatment rescued the cranioskeletal phenotype of *ncl^−/−^* larvae (*n*=45). All experiments were performed three times. Scale bars: 100 µm (B); 70 µm (D). Data are represented as mean±s.d. *P<0.05 (two-tailed, paired Student's *t*-test).

To test whether the addition of FGF8 rescued rRNA synthesis, we examined rRNA transcription in FGF8-treated *ncl^−/−^* embryos, and observed an upregulation in 47S rRNA in these embryos (Fig. 6A). This effect could be the result of FGF8 interacting with rDNA either directly, or through Fgfr2, which has been shown to positively regulate rRNA transcription (Neben et al., 2017). Consistent with the rescued levels of rRNAs, the FGF8-treated *ncl^−/−^* embryos also had fewer TUNEL+ cells compared with *ncl^+/+^* embryos (Fig. 6B), indicating that FGF-mediated rRNA transcription is important for cell survival.

To test whether this initial rescue of apoptosis in FGF8-treated *ncl^−/−^* embryos was enough to rescue the cartilage and bone hypoplasia, we treated *ncl^+/+^* and *ncl^−/−^* embryos with FGF8 at 30 hpf, which is when *ncl^−/−^* embryos no longer exhibit high apoptotic and necrotic tissue (Fig. S8). We observed a similar rescue of the *ncl^−/−^* embryo phenotype at 5 dpf when treatment with FGF8 began at 18 hpf and 30 hpf (Fig. S9), suggesting that exogenous FGF8 improved chondrogenesis and osteogenesis in *ncl^−/−^* embryos.

To analyze whether FGF8 treatment rescued the cranioskeletal phenotype of *ncl^−/−^* mutant embryos via restoration of rRNA synthesis, we treated the *ncl^+/+^* and *ncl^−/−^* embryos at 18 hpf with BMH21 in concert with FGF8. BMH21 is a specific inhibitor of Pol I and dramatically reduces rRNA transcription (Peltonen et al., 2014). With BMH21 treatment alone, the *ncl^+/+^* embryos exhibited a similar loss of cartilage development as seen in *ncl^−/−^* embryos (*n*=45/45), whereas the phenotype of BMH21-treated *ncl^−/−^* embryos remained unchanged (*n*=45/45). The chondrogenic hypoplasia phenotype in BMH21-treated *ncl^+/+^* embryos may have been due to the loss of rRNA synthesis and p53-dependent apoptosis. However, the *ncl^−/−^* embryos already had reduced rRNA synthesis, whereas p53 was increased transiently such that BMH21 treatment did not affect their phenotype. FGF8 plus BMH21 treatment led to severe craniofacial cartilage and bone anomalies in *ncl^+/+^* larvae at 5 dpf, in association with diminished rRNA transcription (*n*=45/45) (Fig. 6D; Fig. S8) and possibly p53-dependent apoptosis and necrosis in the presence of Nucleolin (Fig. S8C,D). However, the cranioskeletal defects in the *ncl^−/−^* larvae were rescued in a manner similar to that seen in the larvae treated with FGF8 alone (*n*=45/45) (Fig. 6D), suggesting that this mechanistically occurs independent of rRNA transcription.

### *ncl^−/−^* mutant embryos are rescued through the Bmp2 signaling pathway

It is well known that the Fgf and Bmp signaling pathways interact synergistically during craniofacial development (Lovely et al., 2016). More specifically, Fgf8 and Bmp2 regulate osteochondroprogenitor differentiation by positively regulating Sox9 expression (Chen et al., 2019; Dash and Trainor, 2020; Shao et al., 2015). Therefore, we examined the expression of *bmp2* mRNA in untreated and FGF8-treated *ncl^−/−^* embryos at 30 hpf. We observed that, whereas *bmp2* mRNA expression was downregulated in untreated *ncl^−/−^* embryos, its expression was increased in FGF8-treated *ncl^+/+^* and *ncl^−/−^* embryos, suggesting that exogenous FGF8 stimulates Bmp signaling in osteochondroprogenitors (Fig. 6C).

Because *bmp2* expression was downregulated in *ncl^−/−^* embryos, but was restored upon FGF8 treatment alone, we hypothesized that the FGF8-based rescue occurs via BMP2 signaling. To test this hypothesis, we treated the *ncl^+/+^* and *ncl^−/−^* embryos with BMP2 alone, and observed a rescue of craniofacial cartilage and bone in *ncl^−/−^* larvae, similar to that seen in embryos with FGF8 treatment alone (*n*=45/45) (Fig. 6D). Furthermore, we quantified rRNA transcription in BMP2-treated embryos and observed downregulation of the 5′ETS and 18S, similar to what was seen in DMSO-treated *ncl^−/−^* embryos at 28 hpf (Fig. S10).

Taken together, our data suggest that Nucleolin regulates craniofacial development through two different pathways, one by controlling rRNA transcription, which appears to be important for cell survival, and the other by post-transcriptionally regulating FGF8 signaling during NCC-derived osteochondroprogenitor differentiation.

## DISCUSSION

Nucleolin is the most abundant phosphoprotein in the nucleolus (Tajrishi et al., 2011); however, its activity and localization are not limited to the nucleolus. Nucleolin has been observed in the nucleus, cytoplasm and plasma membrane, where, depending on the cell type and the environmental condition, Nucleolin performs various functions including DNA replication and repair (Kobayashi et al., 2012), chromatin remodeling (Cong et al., 2012), rRNA transcription and processing (Ginisty et al., 1998; Roger et al., 2003), mRNA turnover and translation (Abdelmohsen et al., 2011; Chen et al., 2000; Fähling et al., 2005; Jiang et al., 2006; Takagi et al., 2005; Zaidi and Malter, 1995; Zhang et al., 2006, 2010) and viral entry and replication (Bose et al., 2004; Izumi et al., 2001; Nisole et al., 2002). Although the cellular and molecular functions of Nucleolin have been previously studied *in vitro*, its role in vertebrate development remains to be explored. Here, we show that Nucleolin is crucial for zebrafish craniofacial development, and specifically for proper differentiation of NCCs into cartilage and bone. Consistent with our findings, Ncl knockdown in *Xenopus* has also been shown to result in craniofacial cartilage hypoplasia (Delhermite et al., 2022).

**Fig. 7.**
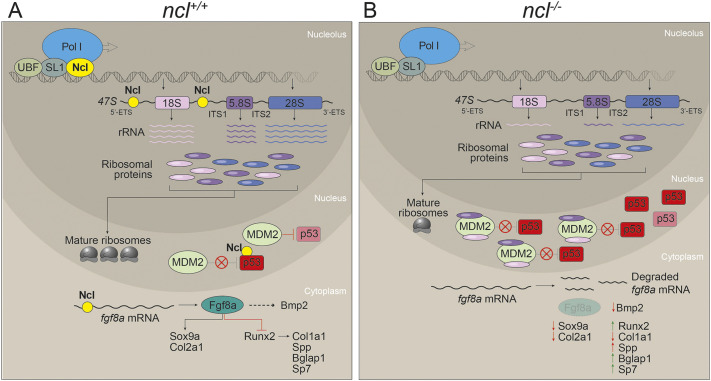
**Nucleolin regulates rRNA transcription and *fgf8a* mRNA stability.** (A) In wild-type zebrafish embryos, Nucleolin is required for rRNA transcription as well as processing. The rRNA transcripts are assembled together with ribosomal proteins to make ribosomes. Meanwhile, Mdm2 binds to and ubiquitylates p53, which results in p53 proteasomal degradation. Nucleolin binds to and stabilizes the p53 protein, thereby acting antagonistically to Mdm2. Furthermore, Nucleolin binds to and stabilizes *fgf8a*, which results in Fgf8a- and Bmp2-regulated chondrogenesis and osteogenesis, leading to proper craniofacial development. (B) In *ncl^−/−^* embryos, the absence of Nucleolin results in reduced rRNA transcription and possibly a free pool of ribosomal proteins that bind to Mdm2. This limits Mdm2 binding to p53, resulting in a temporary increase in p53. However, owing to the lack of Nucleolin in the cell, the p53 protein and *fgf8a* mRNA have reduced half-lives. This results in misregulated chondrogenesis and osteogenesis, leading to cranioskeletal defects.

Similar to other rRNA modifying proteins such as Nol11, Wrd43 and Fibrillarin, the absence of which results in chondrogenesis defects in zebrafish and frog (Bouffard et al., 2018; Griffin et al., 2015; Zhao et al., 2014), it is interesting that perturbation of a global process such as ribosome biogenesis results in craniofacial defects. One possible reason for this tissue specificity could be that the genes required for rRNA transcription, such as those encoding subunits of RNA Pol I, and *fbl* and *ncl*, are highly expressed in NCCs and craniofacial tissues, which makes them more susceptible to disruptions in rRNA transcription (Bouffard et al., 2018; Watt et al., 2016, 2018; Weaver et al., 2015). rRNA transcription is essential not only because rRNA forms the catalytic core of ribosomes, but also because, in its absence, cells undergo proteotoxic stress as a result of ribosomal protein accumulation (Albert et al., 2019; Tye et al., 2019). Under such circumstances, it has been proposed that free ribosomal proteins bind to Mdm2, resulting in the accumulation of p53 and, consequently, cell cycle arrest and cell death (Donati et al., 2011; Lohrum et al., 2003; Scala et al., 2016; Zhang et al., 2003) (Fig. 7A,B).

Contradicting previous *in vitro* studies in human cell lines (Chen et al., 2012), we observed that Nucleolin does not bind to *p53* mRNA *in vivo* in zebrafish, most likely owing to the differences in the 5′ and 3′ UTR sequences of human and zebrafish *p53*. However, the Nucleolin and p53 proteins bind to each other, suggesting that Nucleolin is required to stabilize the p53 protein (Saxena et al., 2006). In the absence of Nucleolin, p53 accumulates temporarily and apoptosis is initiated, however, the effect is not sustained, and it does not result in lethality. This is consistent with an initial upregulation of *p21* mRNA expression from 18 to 24 hpf, followed by a steep decrease by 36 hpf, and is indicative of a momentary surge in the transcriptional activity of p53. p21 is a cell cycle inhibitor, which, when significantly reduced, releases cell cycle arrest (Gartel and Radhakrishnan, 2005; Hemmati et al., 2005), resulting in higher proliferation in *ncl^−/−^* embryos compared with *ncl^+/+^* embryos. The increase in proliferation in the absence of Nucleolin is contrary to published literature, which suggests that Nucleolin promotes proliferation in various cell types (Jiang et al., 2015; Ugrinova et al., 2007). This indicates that a different mechanism regulates proliferation in the absence of Nucleolin in mammalian cells and developing zebrafish. Over time, apoptosis decreases, and proliferation increases in *ncl^−/−^* embryos, which results in embryonic survival until 5 dpf.

Given the ubiquitous expression of Nucleolin in early zebrafish embryos and its requirement in rRNA synthesis, which is essential for ribosome biogenesis and protein translation in all cell and tissue types, we hypothesize that Nucleolin is crucially required during the cleavage, gastrulation and segmentation stages. However, the maternal loading of Nucleolin in the *ncl^−/−^* embryos until 18 hpf is sufficient for survival of the mutant embryos and for the NCCs to form and migrate to the pharyngeal arches. Further studies are required to understand the function of Nucleolin in early NCC development by either generating maternal zygotic *ncl* mutants or establishing an NCC-specific *ncl* conditional deletion mutant. From our current analysis, however, we can conclude that Nucleolin is required for NCC differentiation into chondrocytes and osteocytes in the zebrafish craniofacial region.

Defects in cartilage and bone formation, such as the cartilage hypoplasia, inverted ceratohyal and underdeveloped ceratobranchials, which are observed in *ncl^−/−^* mutants, are common phenotypes typically associated with defects in NCC formation and migration. However, in *ncl^−/−^* mutants, NCC formation and migration to pharyngeal arches appears to be normal, based on lineage-labeling with *sox10:egfp* as well as endogenous Sox10 expression*.* Instead, the chondrogenic hypoplasia phenotype in *ncl^−/−^* mutants can be linked to *sox9a* downregulation, whereas the osteogenic defects arise due to the upregulation of Runx2. This leads to premature osteoblast differentiation, which reduces the pool of cells that can subsequently differentiate into osteoblasts. Thus, early osteoblast markers such as *spp1* and *sp7* are more highly expressed in *ncl^−/−^* embryos compared with their *ncl^+/+^* siblings, while late osteoblast markers such as *bglap* are downregulated. However, both *sox9a* and *runx2* are indirect targets of Nucleolin based on *in silico* analysis of their 5′ and 3′ UTRs, which do not contain a Nucleolin consensus binding site. *fgf8a*, which is also downregulated in *ncl^−/−^* mutants and is required for osteochondroprogenitor differentiation, does contain a Nucleolin-binding site in its 5′ UTR. The 5′ UTR of an mRNA is responsible for both stability and translation of mRNA (Dash et al., 2016). An RNA-binding protein binding to the 5′ UTR of an mRNA has two probable functions: (1) it can stabilize/degrade mRNA; or (2) it can promote/hinder protein translation. Combined with our data that *fgf8a* mRNA decreases over time in the *ncl^−/−^* mutants, this suggests that *fgf8a* is post-transcriptionally regulated by Nucleolin, which results in reduced translation, indicating that Nucleolin must stabilize *fgf8a* mRNA. In addition, the maternal expression of Nucleolin in zebrafish until 18 hpf possibly prevents early embryonic phenotypes such as agenesis of the cerebellum and MHB organizer, which have previously been associated with *fgf8a* downregulation (Reifers et al., 1998). This is further corroborated by the restoration of osteogenesis and chondrogenesis in *ncl^−/−^* mutants with exogenous FGF8, suggesting that the crucial time for Fgf8a function in *ncl^−/−^* zebrafish is around 18 hpf. FGF8 treatment increased the survival of *ncl^−/−^* larvae by 5 days, implying continued treatment may further augment lifespan, and this will be tested in the future.

In mice, Fgf8 has been shown to preferentially bind to Fgfr1 (Mott et al., 2010). However, in zebrafish, Fgf receptors are functionally redundant with respect to Fgf8a ligand binding (Leerberg et al., 2019). Therefore, it is likely that Fgf8a could activate Fgfr2, which is known to bind to the promoter of rDNA, resulting in histone modification and activation of rRNA transcription, similar to Nucleolin (Neben et al., 2017). This suggests that, upon FGF8 treatment, either FGF8 or Fgfr2 translocate to the nucleolus and change the state of the rDNA chromatin from closed to open. However, given that the upstream core elements of rDNA, to which RNA Pol I and UBTF bind, are as yet unannotated in zebrafish, and further work is required to test our hypothesis. This will involve identifying the upstream core elements using a combination of assays for transposase-accessible chromatin using sequencing (ATAC-seq) and chromatin immunoprecipitation followed by sequencing (ChIP-seq), followed by elucidating the mechanism of Fgf8 and Fgfr2 regulation of rRNA transcription in the absence of Nucleolin.

Exogenous FGF8 restores rRNA transcription in *ncl^−/−^* mutants, but the rescue of cranioskeletal defects in *ncl^−/−^* mutants by FGF8 can still occur in the absence of rDNA transcription, as evidenced by BMH21 treatment, possibly because the ribosomal stress-induced p53 response is not sustained in *ncl^−/−^* embryos. This suggests that the effect of FGF8 on osteochondroprogenitor differentiation is mechanistically independent of rRNA synthesis and occurs via its downstream regulation of Bmp2 signaling, which was corroborated by the BMP2-mediated rescue of the cranioskeletal anomalies in *ncl^−/−^* mutants.

Interestingly, *Fgf8* has been previously shown to regulate variance in facial shape in a dose-dependent manner (Green et al., 2017), and rDNA transcription is known to be essential for craniofacial development (Terrazas et al., 2017; Trainor and Merrill, 2014) and variation (Claes et al., 2018). Therefore, as an upstream regulator of *fgf8*, and with a role in rDNA transcription, Nucleolin, by extrapolation, may also play an important role in determining facial shape. Overall, our work has uncovered that Nucleolin regulates Fgf8 signaling as well as rRNA transcription, making craniofacial development especially susceptible to Nucleolin loss of function.

## MATERIALS AND METHODS

### Zebrafish

Adult zebrafish (*Danio rerio*) were housed and maintained in the Stowers Institute Zebrafish Facility according to an Institutional Animal Care and Use Committee (IACUC)-approved protocol (#2021-124). Zebrafish embryos were raised at 28.5°C and staged using standard procedures (Kimmel et al., 1995). To prevent pigment development for immunostaining experiments, 0.002% 1-phenyl-2-thiourea was added to the embryo media. *ncl^hi2078Tg^* zebrafish were obtained through the Zebrafish International Resource Center (ZIRC) and maintained as heterozygotes on the AB/TU background and incrossed to generate homozygous mutant embryos. PCR was used to detect for the presence or absence of the insertional mutation. The *ncl* wild-type allele was detected using the following primers: forward, 5′-TTACATGTGGTGAGAAGGCCC-3′, and reverse, 5′-AACACCTCCCCTGGGTTTAT-3′. The *ncl* mutant allele was detected using the following primers: forward, 5′-TTACATGTGGTGAGAAGGCCC-3′, and reverse, 5′-GCTAGCTTGCCAAACCTACAGGT-3′. The *ncl* heterozygous mutant lines were crossed with the reporter line *Tg(7.2kb-sox10:gfp)*, referred to as *sox10:egfp*, as well as with the *tp53^M214K^* line.

### Live imaging

Embryos were anesthetized with MS-222 (Sigma-Aldrich, A5040) and mounted in 2% methyl cellulose while submerged in E2 media. Embryos were imaged using a Leica MZ16 microscope equipped with a Nikon DS-Ri1 camera and NIS Elements BR 3.2 imaging software. When appropriate, manual *z*-stacks were taken and the images were assembled using Helicon Focus software.

### Skeletal stain

Alcian Blue staining and Alizarin Red staining to label the cartilage and bone, respectively, were performed according to Walker and Kimmel (2007). Embryos were cleared in glycerol and potassium hydroxide and dissected for neurocranium and viscerocranium images. Imaging was performed as described above. Length measurements were performed using ImageJ.

### Immunostaining, EdU and TUNEL

Whole-mount immunostaining was performed according to standard protocols (Westerfield, 2000) using primary antibodies against Nucleolin (1:500, Abcam, ab22758), GFP (1:500, Life Technologies, A6455), Zn-8 (1:250, Developmental Studies Hybridoma Bank), Sox10 (1:500, GeneTex, GTX128374), Sox9a (1:500, GeneTex, GTX128370), Runx2 (1:500, Abcam, ab23981), Fgf8a (1:500, GeneTex, GTX128126), pHH3 (1:2000, Millipore, 06-570). Fluorescent secondary antibodies, either Alexa Fluor 488 or Alexa Fluor 546 (1:500, Invitrogen) were used for detection. The TUNEL and EdU Click-IT assays were performed according to the manufacturer's instructions with slight modifications. Embryos were incubated for 1 h on ice and 1 h at 37°C in the reaction buffer for TUNEL assay. Embryos were imaged using a Zeiss upright 700 confocal microscope, and images were captured and processed using Zen software. ImageJ software was used to quantify the fluorescence intensity and area. The pHH3 and EdU+ cells quantified were in the craniofacial region between the frontonasal prominence and the otic vesicle.

For section staining, embryos were collected at 18 hpf and fixed with 4% paraformaldehyde overnight, followed by equilibration in 30% sucrose overnight. The embryos were then embedded in tissue-freezing media and sectioned at 10 μm thickness. The sections were then immunostained for Nucleolin (1:500, Abcam, ab22758) and imaged using a Zeiss upright 700 confocal microscope.

### *In situ* hybridization

*In situ* hybridization was performed using probes against *runx2a* and *fgf8a* according to standard protocols. Briefly, the embryos were treated with 10 µg/µl proteinase K and hybridized in the probes diluted to 1 ng/μl overnight at 68°C. Following hybridization, the embryos were washed, blocked and incubated with anti-digoxigenin-AP Fab fragments (1:5000, Roche, 11093274910). Signals were detected using nitro blue tetrazolium (NBT)/5-bromo-4-chloro-3-indolyl phosphate (BCIP) and the embryos were imaged using a Nikon DS-Ri1 camera.

### Alkaline phosphatase staining

Fixed embryos were washed in TBS and NTMT buffer (100 mM NaCl, 100 mM Tris-HCl pH 9.5, 50 mM MgCl_2_, 1% Tween 20), followed by incubation with NBT (3.5 µl) and BCIP (5 µl) in NTMT for the desired color revelation time. The images were collected using a Nikon DS-Ri1 camera.

### Western blotting

Protein samples consisting of five fish/sample were collected at appropriate stages. Embryos were homogenized and suspended in sample buffer containing Tris-HCl pH 8.0, NaCl, SDS, sodium deoxycholate, NP-40 and protease inhibitor and used for western blotting according to standard protocols (Dash et al., 2021). Protein quantity was estimated via a bicinchoninic acid (BCA) assay. The primary antibodies used were: γ-tubulin (1:1000, Sigma-Aldrich, T6557), α-tubulin (1:10,000, Sigma-Aldrich, T5168), p53 (1:500, Cell Signaling Technology, 2524S) and Nucleolin (1:500, Abcam, ab22758). Western blots were imaged and quantified using a CLx-Scanner (Li-COR) and Odyssey Software. For quantification, band intensities for p53 were compared with the housekeeping control protein γ-tubulin. Two-tailed, paired Student's *t*-test was performed for statistical analysis.

### Immunoprecipitation

Protein lysates from 28 hpf control and *ncl^−/−^* zebrafish were used (*n*=25 per biological replicate; total three biological replicates) for pre-conjugation with the p53 antibody (Cell Signaling Technology, 2524S, 1:100) and IgG with magnetic beads. The same number of embryos were used for each group. Equal volumes of the immunoprecipitates were then loaded on two SDS-PAGE gels and transferred to polyvinylidene difluoride (PVDF) membranes to immunoblot for Mdm2 (1:1000, Cell Signaling Technology, 86934) and p53 (1:1000, Cell Signaling Technology, 2524) separately. Because the embryos were grouped and lysed immediately after collection, genotyping by conventional methods could not be performed. Instead, the lysate was used in a western blot for Nucleolin to substitute as genotyping. Following immunoprecipitation, the samples were used in western blotting and immunoblotted for both Mdm2 and p53.

### qPCR

For sample collection, embryos were genotyped individually using the tip of the tail tissue at the required developmental stage. After the identification of mutants and wild types from a clutch, ten embryos of the same genotype were pooled together for RNA isolation (*n*=1). RNA was collected from zebrafish embryos using the Qiagen miRNeasy Micro Kit and tested for quality on an Agilent 2100 Bioanalyzer. The Superscript III kit (Invitrogen) was used to synthesize cDNA for qPCR using random hexamer primers. The following primers were used: *ncl* forward, 5′-ATATCGAGGGCAGGAGTATT-3′, and reverse, 5′-GTTTTCGTAGGTCCAGAGTT-3′; *tp53* forward, 5′-CGAGCCACTGCCATCTATAAG-3′, and reverse, 5′-TGCCCTCCACTCTTATCAAATG-3′; *p21* forward, 5′-GACCAACATCACAGATTTCTAC-3′, and reverse, 5′-TGTCAATAACGCTGCTACG-3′; *sox9a* forward, 5′-GGAGCTCAGCAAAACTCTGG-3′, and reverse, 5′-AGTCGGGGTGATCTTTCTTG-3′; *col2a1* forward, 5′-GCGACTTTCACCCCTTAGGA-3′, and reverse, 5′-TGCATACTGCTGGCCATCTT-3′; *runx2a* forward, 5′-AACTTTCTGTGCTCGGTGCT-3′, and reverse, 5′-AACTTTCTGTGCTCGGTGCT-3′; *runx2b* forward 5′-CAAACACCCAGACCCTCACT-3′, and reverse, 5′-GTATGACCATGGTGGGGAAG-3′; *col1a2* forward, 5′-CTGGCATGAAGGGACACAG-3′, and reverse, 5′-GGGGTTCCATTTGATCCAG-3′; *col10a1a* forward, 5′-CCTGTCTGGCTCATACCACA-3′, and reverse, 5′-AAGGCCACCAGGAGAAGAAG-3′; *fgf8a* forward, 5′-GCCGTAGACTAATCCGGACC-3′, and reverse, 5′-TTGTTGGCCAGAACTTGCAC-3′; *bglap* forward, 5′-TGAGTGCTGCAGAATCTCCTAA-3′, and reverse, 5′-GTCAGGTCTCCAGGTGCAGT-3′; *spp1* forward, 5′-TGAAACAGATGAGAAGGAAGAGG-3′, and reverse, 5′-GGGTAGCCCAAACTGTCTCC-3′; *sp7* forward, 5′-GGATACGCCGCTGGGTCTA-3′, and reverse, 5′-TCCTGACAATTCGGGCAATC-3′; *bmp2* forward, 5′-TCCATCACGAAGAAGCCGTGG-3′, and reverse, 5′-TGAGAAACTCGTCACTGGGGA-3′; *actb* forward, 5′-TTCCTTCCTGGGTATGGAATC-3′, and reverse, 5′-GCACTGTGTTGGCATACAGG-3′; and *canx* forward, 5′-ACGATACCGCAGAGAATGGAGACA-3′, and reverse, 5′-TCCTGTTTCTGGGAGACCTCCTCA-3′. Previously published rRNA primer sequences were used for qPCR (Azuma et al., 2006). Power Sybr (Life Technologies) reaction mix and the ABI 7900HT real time PCR cycler was used to measure cDNA amplification. Three biological replicates were run in technical triplicate for each experiment. Samples without the template and without reverse transcriptase were run as negative controls.

### RNA immunoprecipitation

Lysates from 28 hpf wild-type zebrafish were used (*n*=100 per biological replicate; total three biological replicates) for pre-conjugation with the Nucleolin antibody (Abcam, ab22758, 1:100) and IgG with magnetic beads. Immunoprecipitation was performed using the manufacturer's instructions for the EZ-Nuclear RIP Kit (EMD Millipore, 17-10523), followed by cDNA synthesis and qRT-PCR for 5′ETS, ITS1, ITS2, 18S rRNA, *fgf8a*, *p53* and *actb*.

### Drug treatments

*ncl^+/+^* and *ncl^−/−^* embryos were treated with 0.0625, 0.25 and 1 ng/μl human recombinant FGF8 (Thermo Fisher Scientific, PHG0184) as well as 1 ng/μl FGF8 along with 2 ng/μl BMH21 (Sigma-Aldrich, SML1183) and 1 μg/μl BMP2 (Stemcell Technologies, 78004) diluted in E2 media by immersion starting at 18 hpf. In addition, *ncl^+/+^* and *ncl^−/−^* embryos were treated with 1 ng/μl FGF8 by immersion beginning at 30 hpf. The drugs in the media were replaced every 24 h until 5 dpf. The FGF8-treated larvae were added to the tank system and allowed to develop until 15 dpf.

### Sample size and statistics

For brightfield imaging and skeletal stain, 15 embryos of each genotype for a particular stage were assayed from one clutch of embryos, and this was repeated three times to ensure adequate power for statistical analysis. For immunostaining, western blotting and drug treatments, five embryos per genotype from a clutch were assayed and analyzed. For qPCR, immunoprecipitation and RNA immunoprecipitation, 10, 25 and 100 embryos per clutch were used, respectively. All experiments were repeated three times to ensure reproducibility. The embryos were chosen at random from a clutch size of at least 200 embryos. None of the data collected were excluded from analysis. For phenotypic analysis, two-tailed, unpaired *t*-test with Welch's modification was performed. For molecular quantifications (western blot, RNA immunoprecipitation and immunoprecipitation), two-tailed, paired *t*-test was used. Two-way ANOVA was performed for statistical analysis of qPCR data. *P*-values for all experiments are provided in Table S1.

## Supplementary Material

Supplementary information

Reviewer comments
