# Correction: Nucleolin loss of function leads to aberrant Fibroblast Growth Factor signaling and craniofacial anomalies

**DOI:** 10.1242/dev.201584

**Published:** 2023-03-27

**Authors:** Soma Dash, Paul A. Trainor

There were errors in *Development* (2022) **149**, dev200349 (doi:10.1242/dev.200349).

In the Materials and Methods section, the *bmp2* primer sequences were listed incorrectly. The corrected text is shown below.

**Corrected:**

The following primers were used:…bmp2 forward, 5′-AGCTTCCACCATGATGAATCTACA-3′, and reverse, 5′-TCAGGTTGAAGAGGAACCGC-3′…

**Original:**

The following primers were used:…bmp2 forward, 5′-TCCATCACGAAGAAGCCGTGG-3′, and reverse, 5′-TGAGAAACTCGTCACTGGGGA-3′…

The authors apologise for these errors and any inconvenience they may have caused. They do not impact the results or conclusions of this article.

Both the online full-text and PDF versions of the paper have been corrected.

