# Supplementary material for: Nucleolin loss of function leads to aberrant Fibroblast Growth Factor signaling and craniofacial anomalies
Source: Development. 2022 Jun 28;149(12):dev200349. doi: 10.1242/dev.200349 (PMC9270975; doi:10.1242/dev.200349)
Supplement: Supplementary information [file develop-149-200349-s1.pdf]

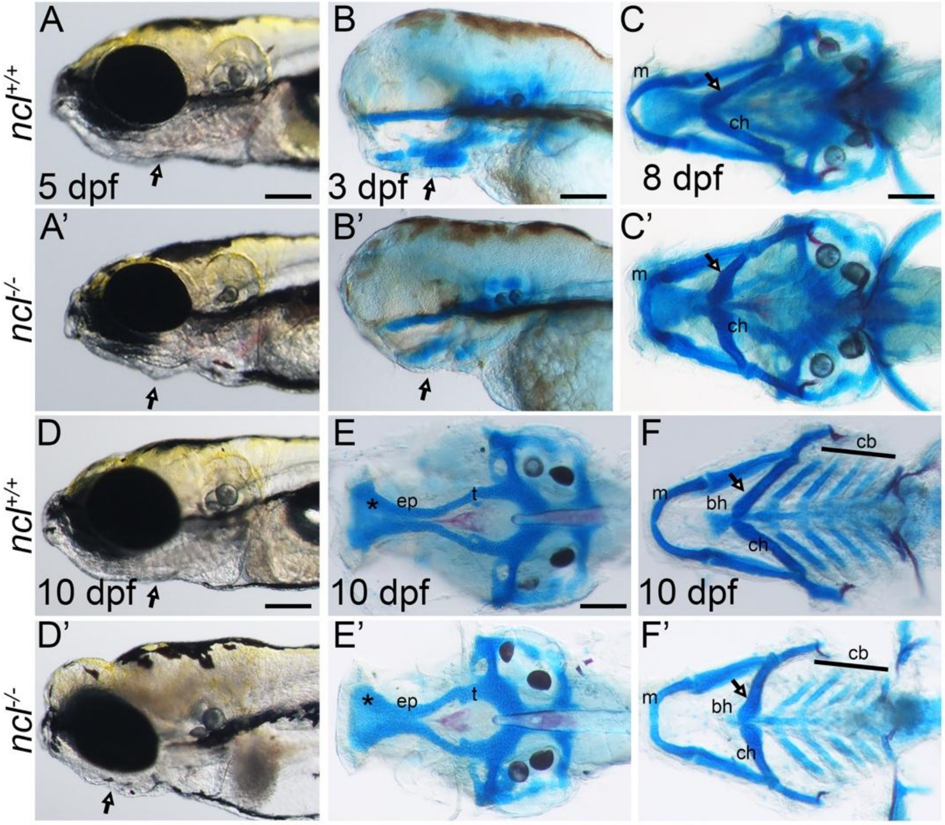

Fig. S1. *ncl$^{-/-}$* mutants exhibit craniofacial defects and embryonic lethality. (A-A') Brightfield imagining of 5 dpf *ncl$^{+/+}$* and *ncl$^{-/-}$* reveals the lower jaw is smaller in *ncl$^{-/-}$* larvae (black arrow). Skeletal staining of 3 dpf (B-B') and 8 dpf (C-C') *ncl$^{+/+}$* and *ncl$^{-/-}$* larvae shows the ceratohyal in hypoplastic in 3 dpf mutants (B') and misshapen in 8 dpf mutants (C'). (D-D') Brightfield imagining of 10 dpf *ncl$^{+/+}$* and *ncl$^{-/-}$* larvae indicates the lower jaw is smaller in *ncl$^{-/-}$* larvae (black arrow) and reveals necrotic tissue in the craniofacial region and the heart. Skeletal staining with Alcian blue shows differential labeling of the ethmoid plate (asterisk), with smaller trabecula in the neurocranium in 10 dpf *ncl$^{-/-}$* larvae compared to controls (E-E'). Furthermore, in the viscerocranium, Meckel's cartilage is bent, the basihyal and ceratobranchials are hypoplastic and the angle of the ceratohyal is obtuse (F-F'). n = 25 per panel. Abbr. ep: ethmoid plate, t: trabecula, m: meckel's, bh: basihyal, ch: ceratohyal, cb: ceratobranchial. Scale bars denotes 350 μm for A-A', 50 μm for B-B', 100 μm for C-F'.

Development • Supplementary information

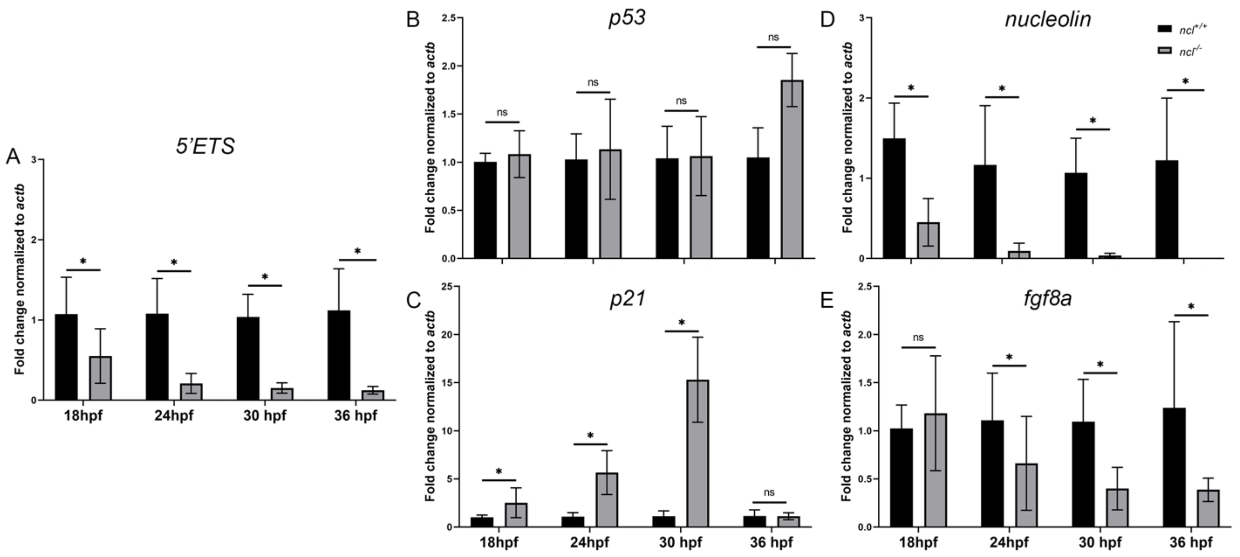

Fig. S2. ncl$^{-/-}$ mutants have lower rRNA and *fgf8a* mRNA expression. qPCR using craniofacial tissues from *ncl*$^{+/+}$ and *ncl*$^{-/-}$ embryos at 18, 24, 30 and 36 hpf indicates that rRNA (A), *ncl* (D) and *fgf8a* (E) expression is significantly downregulated, while *p53* (B) mRNA is unaffected and *p21* (C) mRNA is momentarily upregulated in *ncl*$^{-/-}$ compared to *ncl*$^{+/+}$ embryos (n=10 per sample).

Development • Supplementary information

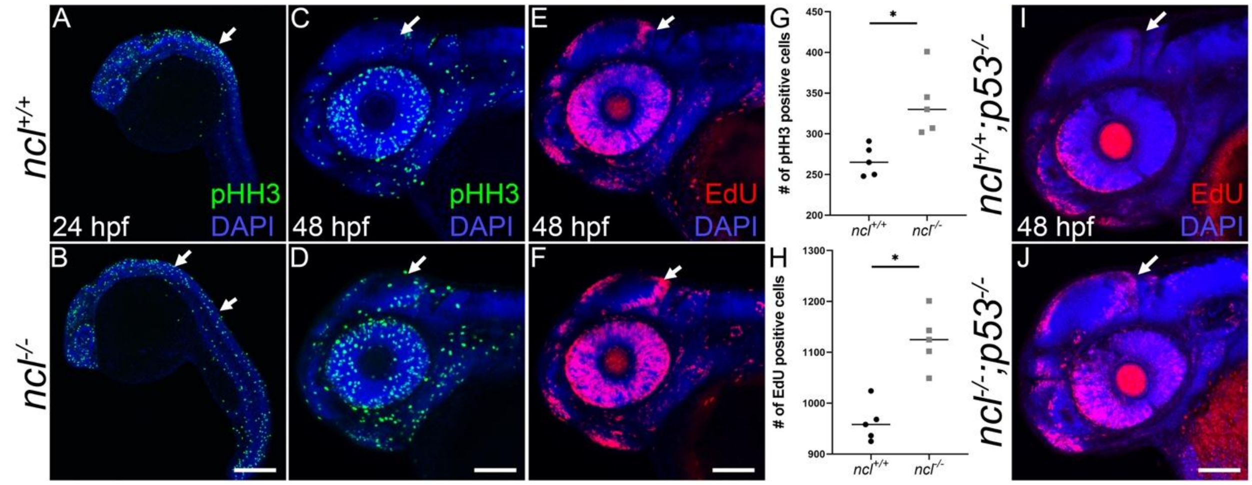

Fig. S3. Proliferation is increased in *ncl*$^{-/-}$ embryos. At 24 hpf, proliferating cells were labeled with the mitotic marker, pHH3, which revealed no significant difference between *ncl*$^{+/+}$ (A) and *ncl*$^{-/-}$ (B) embryos (n =15). By 48 hpf, the number of proliferating cells labeled with pHH3 (C-D) and EdU (E-F) is significantly higher in the *ncl*$^{-/-}$ embryos compared to *ncl*$^{+/+}$ embryos, especially in the midbrain-hindbrain boundary. Quantification of pHH3 (n=5) (G) and EdU (n=5) (H) positive cells in the craniofacial region from frontonasal prominence to the otic vesicle of *ncl*$^{+/+}$ and *ncl*$^{-/-}$ embryos. Similarly, *ncl*$^{-/-}$;*p53*$^{-/-}$ embryos (J) have more proliferating cells than *ncl*$^{+/+}$ embryos (n=15) (I). Scale bar denotes 70 μm for A-F, I-J.

Development • Supplementary information

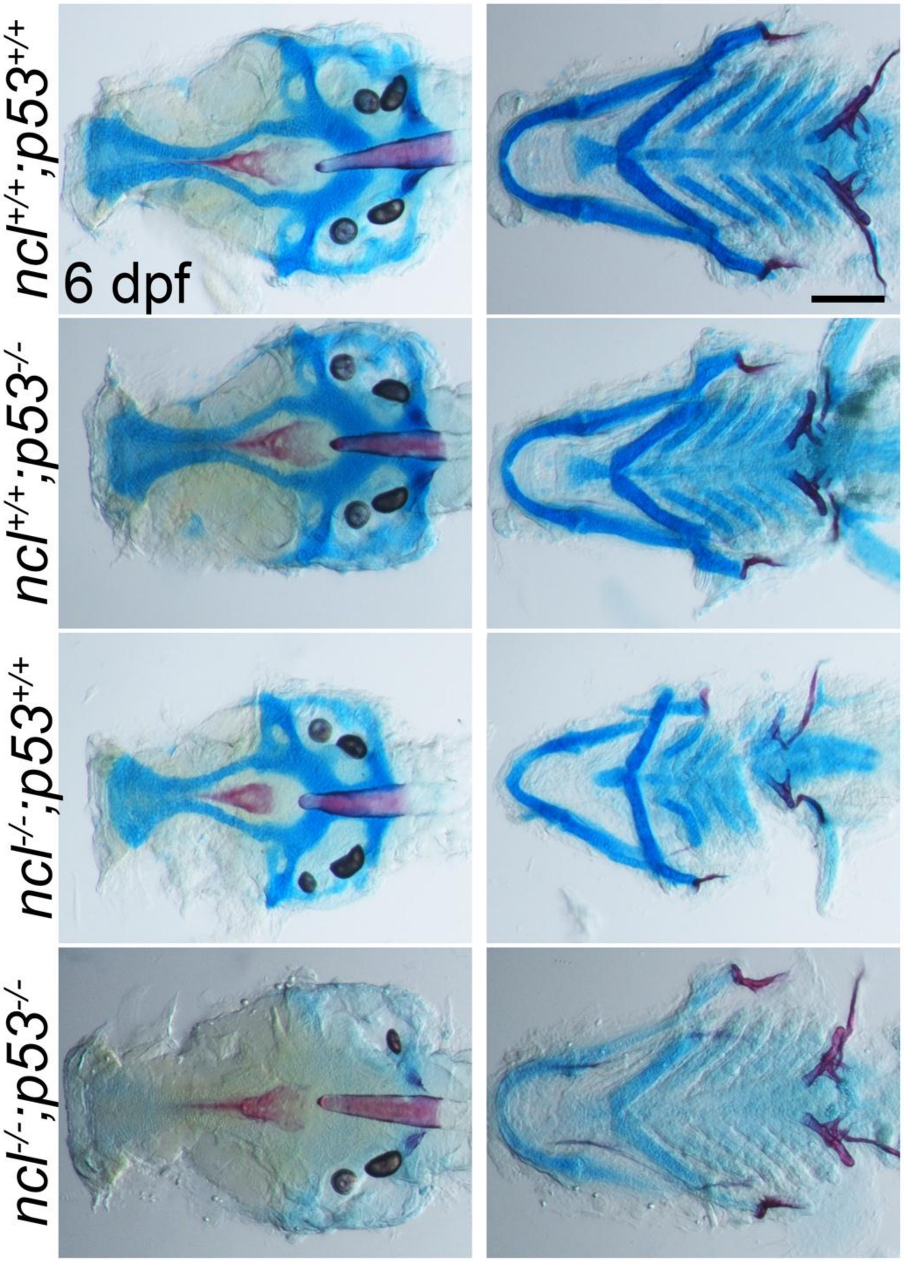

Fig. S4. p53 downregulation does not rescue the cranioskeletal anomalies in *ncl$^{-/-}$* mutants. Compared to *ncl$^{+/+}$;p53$^{+/+}$*, *ncl$^{+/+}$;p53$^{-/-}$* and *ncl$^{-/-}$;p53$^{+/+}$* larvae at 5 dpf, *ncl$^{-/-}$;p53$^{-/-}$* have hypoplastic neurocranium and ceratohyal cartilages. Scale bar denotes 70 μm for A-B.

Development • Supplementary information

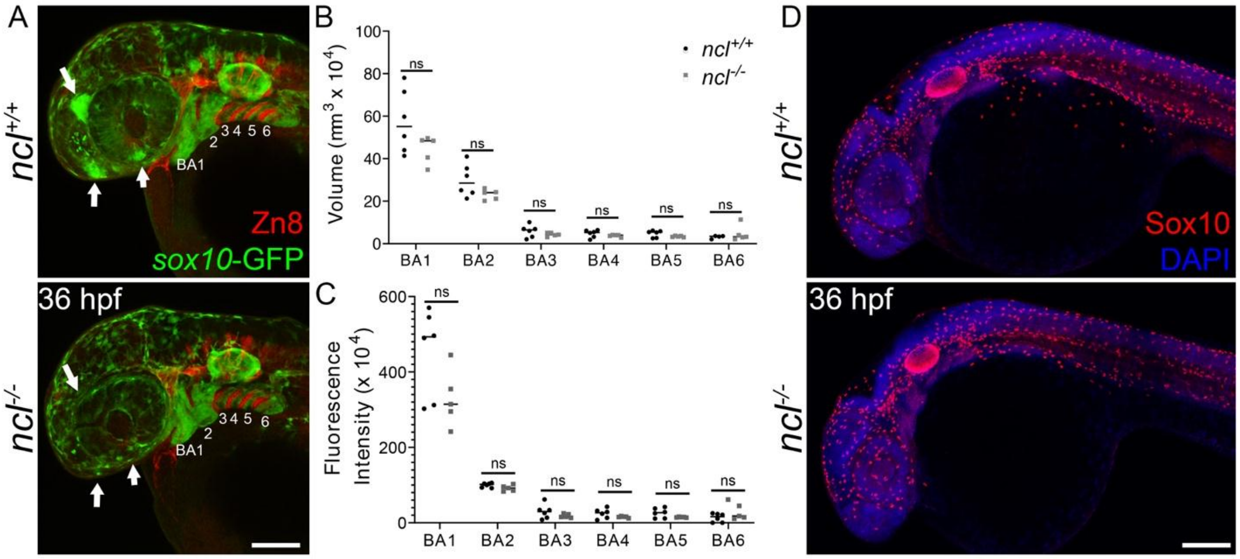

Fig. S5. Neural crest cell induction and migration is unaffected in *ncl*$^{-/-}$ mutant embryos. (A) *ncl*$^{+/+}$ and *ncl*$^{-/-}$ embryos (n=15) at 36 hpf were immunostained with YFP to reveal NCC labeled by the *sox10-egfp* transgene, and for the pharyngeal pouch marker, Zn8 to demarcate each pharyngeal arch. Volumetric (B) and fluorescence intensity (C) analysis of the pharyngeal arches indicates that the arches in *ncl*$^{+/+}$ and *ncl*$^{-/-}$ embryos are of comparable volume and have similar amounts of NCC. (D) Sox10 immunostaining reveals similar numbers of Sox10 positive cells in *ncl*$^{+/+}$ and *ncl*$^{-/-}$ embryos (n=15). Scale bar denotes 100 μm for A and D.

Development • Supplementary information

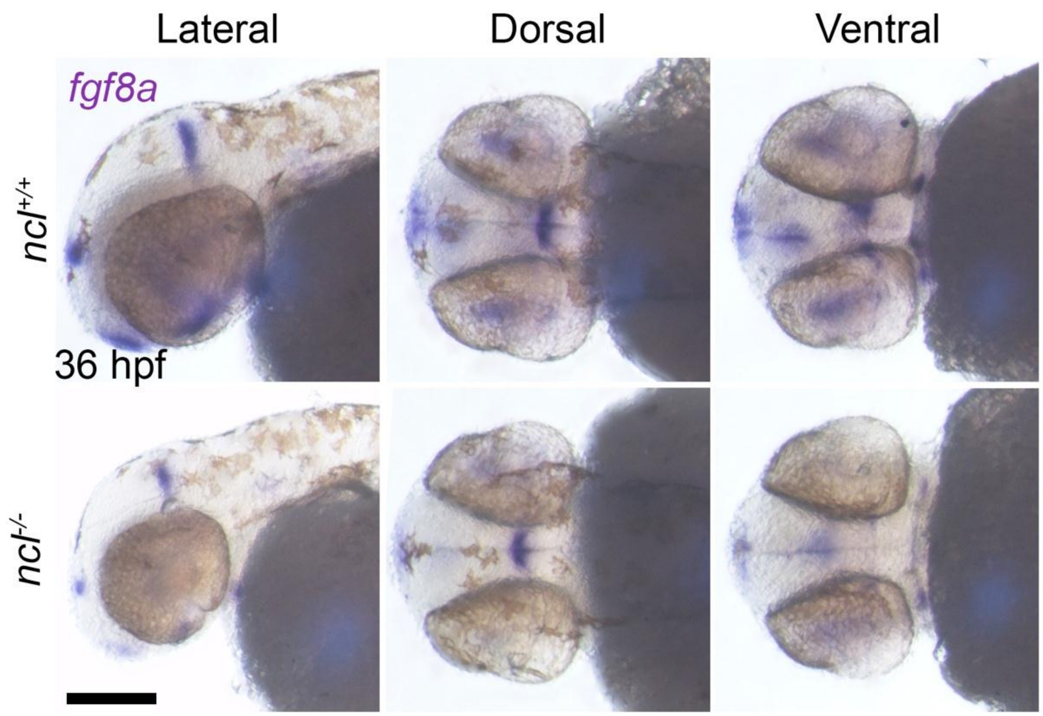

Fig. S6. *fgf8a* is downregulated in *ncl*$^{-/-}$ mutant embryos. *ncl*$^{+/+}$ and *ncl*$^{-/-}$ embryos (n=15) at 36hpf were labeled for *fgf8a* mRNA through in situ hybridization, which shows a reduction in the general expression of *fgf8a*. Scale bar denotes 100 μm.

Development • Supplementary information

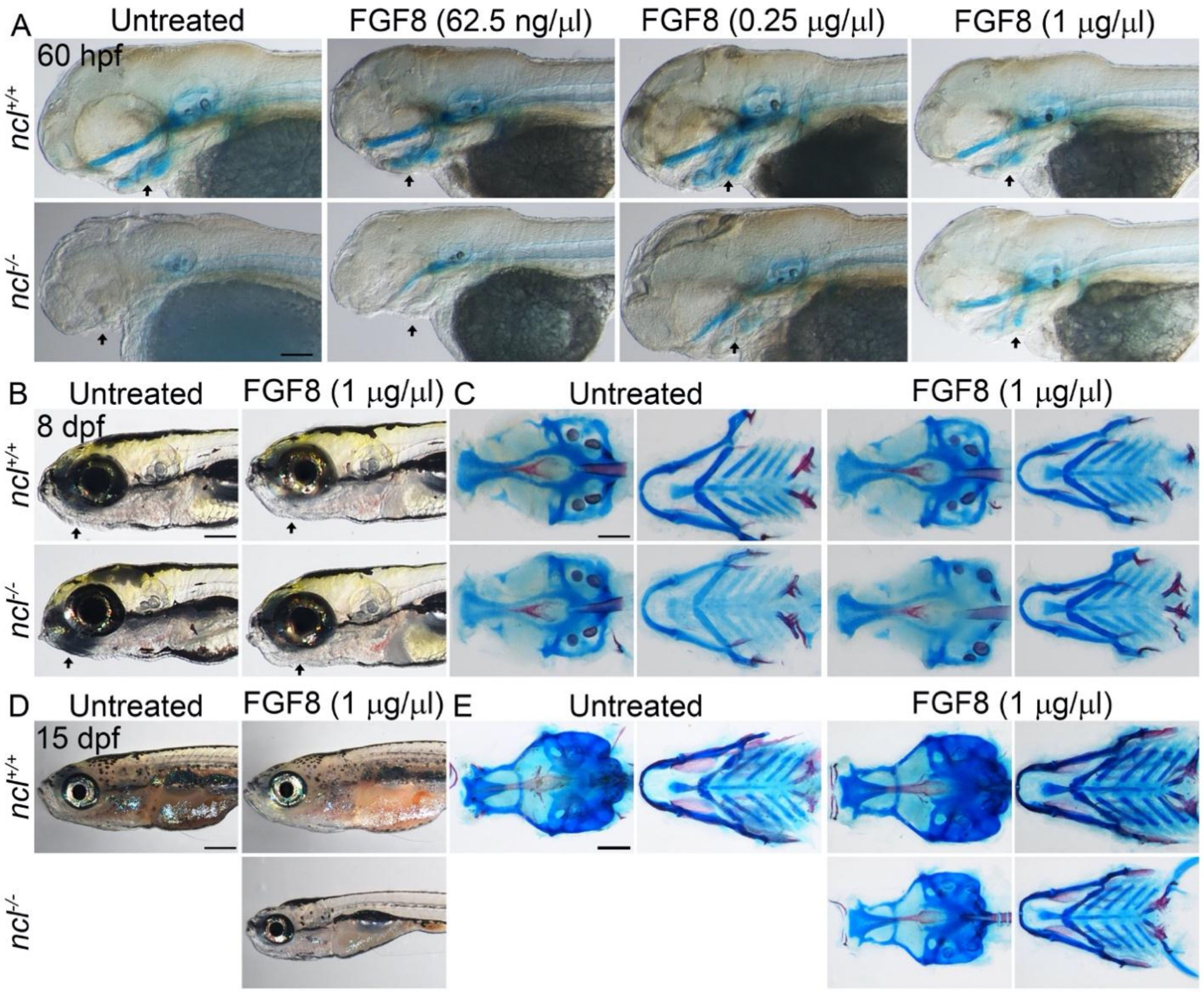

Fig. S7. FGF8 treatment rescues *ncl*$^{-/-}$ embryos. (A) Alcian blue and Alizarin red staining of *ncl*$^{+/+}$ and *ncl*$^{-/-}$ embryos treated with FGF8 indicates that with increasing dose of FGF8, the cartilage elements of 60hpf *ncl*$^{-/-}$ embryos are progressively rescued. (B) Brightfield images of 8 dpf *ncl*$^{+/+}$ and *ncl*$^{-/-}$ larvae show that with FGF8 treatment, the craniofacial and swim bladder phenotypes are rescued. (C) Skeletal staining of 8 dpf untreated and treated *ncl*$^{+/+}$ and *ncl*$^{-/-}$ larvae indicates that most of the cranioskeletal defects including the hypoplastic basihyal and cetatobranchial are rescued with 1 μg/μl FGF8 treatment. (D) Brightfield images of 15 dpf *ncl*$^{+/+}$ and *ncl*$^{-/-}$ larvae show that with FGF8 treatment the larvae are smaller compared to untreated and treated *ncl*$^{+/+}$ larvae. In addition, the anterior swim bladder is not inflated in the treated *ncl*$^{-/-}$

larvae. The untreated *ncl*$^{-/-}$ larvae do not survive beyond 10 dpf. (E) Skeletal staining of 15 dpf untreated and treated *ncl*$^{+/+}$ and *ncl*$^{-/-}$ larvae indicates that most of the cranioskeletal defects are comparable between treated *ncl*$^{+/+}$ and *ncl*$^{-/-}$ larvae, although the embryo is smaller (n=15 per panel). Scale bar denotes 50 μm for A, 250 μm for B, 100 μm for C, 350 μm for D and 200 μm for E.

Development • Supplementary information

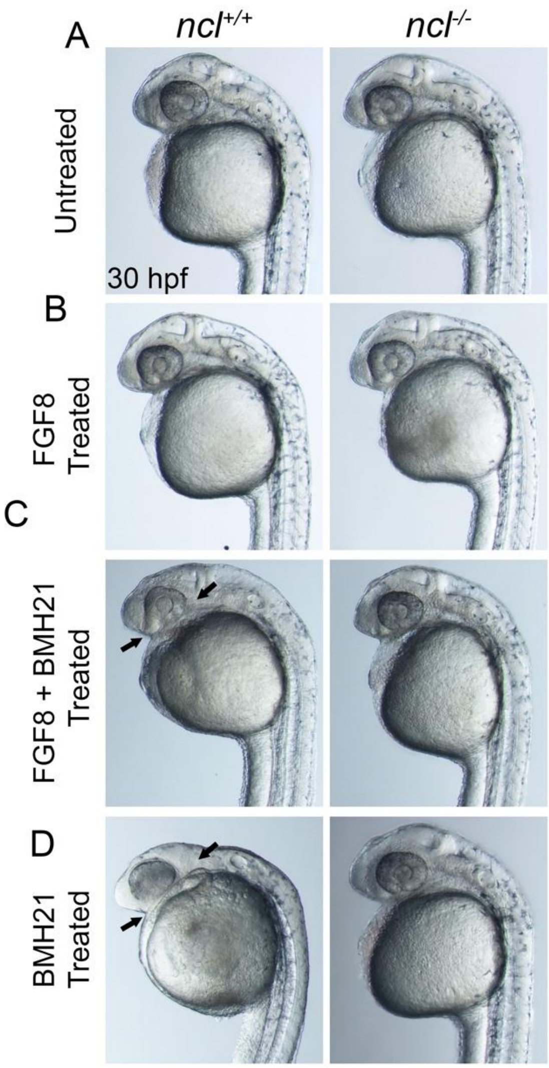

Fig. S8. BMH21 treatment results in apoptosis and necrosis in WT embryos. Bright field images of 30 hpf *ncl*$^{+/+}$ and *ncl*$^{-/-}$ embryos treated with FGF8+BMH21 (C) and BMH21 (D) indicates that BMH21 treatment with or without FGF8 leads to necrosis in *ncl*$^{+/+}$ embryos while *ncl*$^{-/-}$ embryos are phenotypically similar to untreated (A) and FGF8 (B) treated conditions. Drug treatments were started at 18hpf (n=15 per genotype per treatment).

Development • Supplementary information

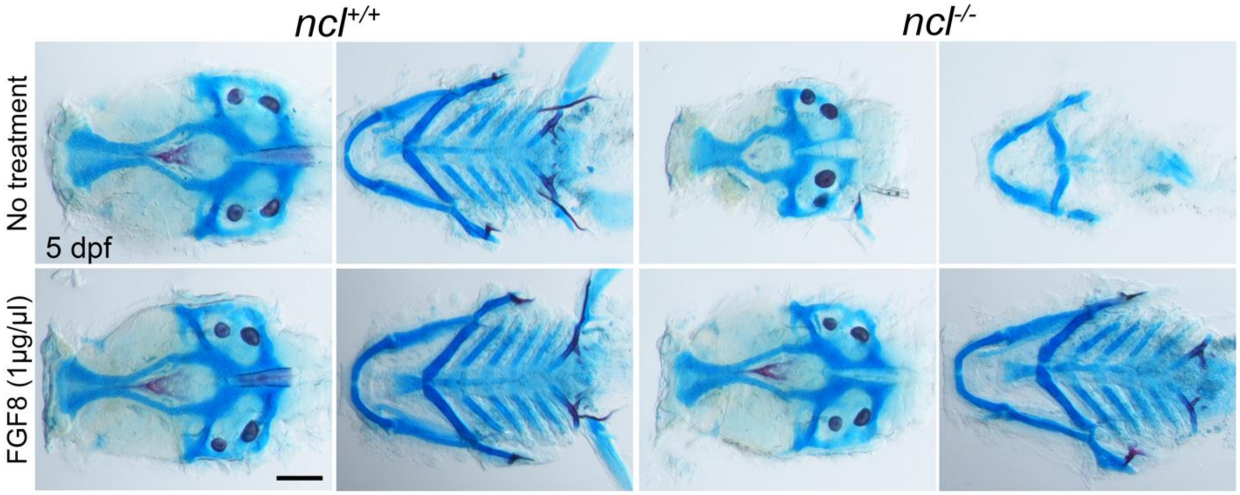

Fig. S9. FGF8 treatment rescues *ncl*$^{-/-}$ embryos. Alcian blue and Alizarin red staining of *ncl*$^{+/+}$ and *ncl*$^{-/-}$ embryos treated with FGF8 at 30hpf indicates that the cartilage and bone elements at 5dpf *ncl*$^{-/-}$ embryos are rescued (n=15 per genotype per treatment).

Development • Supplementary information

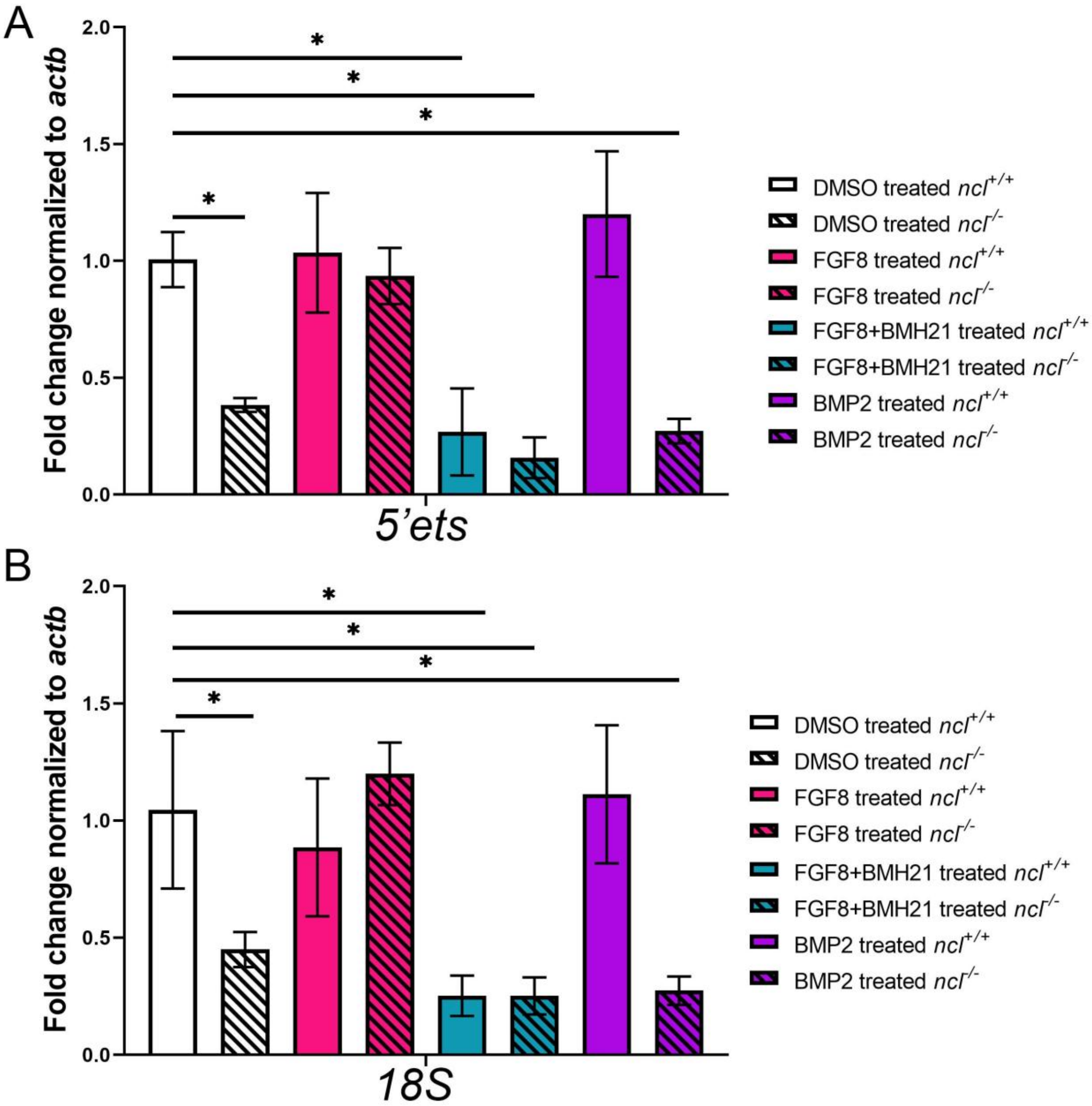

Fig. S10. FGF8 treatment along with BMH21 reduces rRNA transcription. (A-B) qPCR for *5'ets* and *18S* in DMSO, FGF8, FGF8 plus BMH21 and BMP2 treated *ncl*$^{+/+}$ and *ncl*$^{-/-}$ zebrafish (n=10 per sample) indicates rescue of pre-RNA transcription in FGF8 treated *ncl*$^{-/-}$ zebrafish at 28 hpf, while no rescue is observed in FGF8 plus BMH21 and BMP2 treated *ncl*$^{-/-}$ zebrafish. The *5'ets* and *18S* transcript levels were compared to DMSO treated *ncl*$^{+/+}$ zebrafish.

Table S1. List of p-values for qPCR, RIP, IP and immunoprecipitation quantification experiments

Click here to download Table S1
