# Supplementary material for: Nucleolin loss of function leads to aberrant Fibroblast Growth Factor signaling and craniofacial anomalies
Source: Development. 2022 Jun 28;149(12):dev200349. doi: 10.1242/dev.200349 (PMC9270975; doi:10.1242/dev.200349)
Supplement: Reviewer comments [file develop-149-200349_review_history.pdf]

# Nucleolin loss of function leads to aberrant Fibroblast Growth Factor signaling and craniofacial anomalies

Soma Dash and Paul A. Trainor
DOI: 10.1242/dev.200349

**Editor**: Patrick Tam

**Review timeline**

| | |
|---|---|
| Original submission: | 9 November 2021 |
| Editorial decision: | 16 December 2021 |
| First revision received: | 18 March 2022 |
| Editorial decision: | 7 April 2022 |
| Second revision received: | 21 April 2022 |
| Accepted: | 25 April 2022 |


---

**Original submission**

First decision letter

MS ID#: DEVELOP/2021/200349

MS TITLE: Nucleolin loss-of-function leads to aberrant FGF signaling and craniofacial anomalies

AUTHORS: Soma Dash and Paul Trainor

I have now received all the referees' reports on the above manuscript, and have reached a decision. The referees' comments are appended below, or you can access them online: please go to BenchPress and click on the 'Manuscripts with Decisions' queue in the Author Area.

As you will see, the referees express considerable interest in your work, but have some significant criticisms and recommend a substantial revision of your manuscript before we can consider publication. If you are able to revise the manuscript along the lines suggested, which may involve further experiments, I will be happy receive a revised version of the manuscript. Your revised paper will be re-reviewed by one or more of the original referees, and acceptance of your manuscript will depend on your addressing satisfactorily the reviewers' major concerns. Please also note that Development will normally permit only one round of major revision.

We are aware that you may be experiencing disruption to the normal running of your lab that make experimental revisions challenging. If it would be helpful, we encourage you to contact us to discuss your revision in greater detail. Please send us a point-by-point response indicating where you are able to address concerns raised (either experimentally or by changes to the text) and where you will not be able to do so within the normal timeframe of a revision. We will then provide further guidance. Please also note that we are happy to extend revision timeframes as necessary.

Please attend to all of the reviewers' comments and ensure that you clearly highlight all changes made in the revised manuscript. Please avoid using 'Tracked changes' in Word files as these are lost in PDF conversion. I should be grateful if you would also provide a point-by-point response detailing how you have dealt with the points raised by the reviewers in the 'Response to Reviewers' box. If you do not agree with any of their criticisms or suggestions please explain clearly why this is so.

© 2022. Published by The Company of Biologists under the terms of the Creative Commons Attribution License (https://creativecommons.org/licenses/by/4.0/).

Reviewer 1

*Advance summary and potential significance to field*

A recurring phenomenon in neural crest (NC) biology is that NC cells are disproportionately impaired by mutation of genes presumed to be of ubiquitous expression and function, e.g., those involved in rRNA transcription and ribosome biogenesis. Numerous hypotheses have been put forward to explain this curiosity and it has been of interest to the Trainor lab for some time. In this manuscript, Dash & Trainor add nucleolin to this list of ubiquitous genes that NC cells are hypersensitive to the loss of. Ncl is a phosphoprotein that is a major component of the nucleolus (but also expressed in the cytoplasm) and has documented roles in rRNA processing and mRNA/DNA binding. Using an existing zebrafish insertional mutant allele for ncl, the authors show a clear downregulation of rRNA transcription and increased levels of p53 protein/apoptosis in mutant embryos, as well mild craniofacial malformations and lethality. They are able to rescue most of these phenotypes by mutating p53 or by treating with recombinant FGF8 or BMP2, supporting the conclusion that the ncl mutant is suffering from a mild neurocristopathy.

*Comments for the author*

**Major comments**

1. The *ncl* mutant craniofacial phenotype closely resembles the common "sickly" facial development phenotype associated with generally impaired development or morpholino-associated toxicity (hypoplastic cartilages, inverted ceratohyal, poorly stained and perpendicular ceratobranchials, small eyes, misshapen head, uninflated swim bladder). It is not surprising that a mild neurocristopathy caused by mutation in a housekeeping gene like *ncl* would take this form, or that it would be so readily rescued by treatments that counteract cell death. The authors should place this overall phenotype in the context of others in the literature.

2. The authors observe that Ncl protein is maternally supplied, ubiquitously expressed (Fig. 1), and not significantly depleted in mutants until after 12 hpf. Transcript levels in mutants are likewise similar to controls at 18 hpf and only appear to decline (to ~50% of control level) after this stage (Fig. 5B). This issue of maternal loading and the timing of decline warrants more attention. As the zygotic mutants are not adult-viable, I recognize that it is not particularly simple to create a maternal-zygotic mutant to determine how essential this maternal contribution is. However, whether the NCCs are specifically impacted because an earlier/broader requirement for the gene is rescued by the maternal contribution could be discussed in more detail.

3. It is unclear when the rRNA qPCR experiment shown in Fig. 3A was performed, and whether the effect changes over time. If it is prominent early and then normalized through an unknown mechanism, that would support (in part) the authors' model that Nucleolin's critical time of function is in the early pharyngula stages.

4. Ncl has previously been shown to modify p53 levels through both direct and indirect mechanisms. Mutants show unaltered p53 transcript levels but a peak in protein accumulation at the stage (~24 hpf) when cell death is elevated. The authors show that Ncl does not bind p53 mRNA in this context but does interact with the protein in wild-type embryos (stage not noted). However, because p53 protein levels are transiently *increased* in mutants, this result would argue against Ncl contributing to p53 stability. The author then did a rather confusing experiment wherein they performed immunoprecipitation for the p53 ubiquitinator Mdm2 and then blotted simultaneously for Mdm2 and p53, in the same color. The blotting result is interpreted as supposedly decreased pulldown of both Mdm2/p53 in mutants, supposedly meaning that their interaction level is lower, which in turn could explain why p53 levels are transiently higher. This conclusion is problematic on multiple levels - the IP blot in Fig. 2H is smeared and impossible to interpret; less Mdm2 on the mutant blot (if true) would imply less Mdm2 protein pulled down, not less Mdm2-p53 interaction; it is not clear whether p53 protein levels are still elevated at 28 hpf, when the IP was done. The authors' conclusions that "the initial accumulation of p53 is a result of reduced interactions between Mdm2 and p53" and

© 2022. Published by The Company of Biologists under the terms of the Creative Commons Attribution License (https://creativecommons.org/licenses/by/4.0/). 

"The subsidence of p53 activity at later stages might therefore be due to a lack of Nucleolin stabilization of p53 protein" do not seem fully justified by the data presented.

5. The p53 rescue looks quite robust, though the authors should include a picture of an *ncl-/-; p53+/+* larva in Fig. S3A to show that the single mutant phenotype is the same as reported in the main text on this genetic background. Also, weak Alcian staining of ceratohyal chondrocytes is a fairly common staining artifact and should be interpreted with caution. It goes too far to say that these dKOs "die around 10-12 dpf due to these cranioskeletal defects" and that "the skeletal defects in *ncl-/-* mutant zebrafish are not p53 dependent" – they are quite well rescued!

6. *sox10* mRNA is expressed in migrating NCCs and non-ectomesenchymal lineages but is rapidly downregulated ~18 hpf when ectomesenchyme-fated NCCs arrive in the pharyngeal region. It is then re- expressed in differentiating chondrocytes after ~56 hpf. I am thus suspicious of the hazy immunostaining in the arches at 36 hpf (Fig. S4D) – the punctate nuclear staining elsewhere in the body looks real but the arch staining does not.

7. The authors present qPCR data showing that *sox9a* and *col2a1a* are downregulated in mutants [at an undefined stage] and that Sox9a protein levels look slightly lower in the posterior arches at 36 hpf. However, because they appear to have used whole embryos for the RNA extraction/qPCR experiment and because these genes are expressed elsewhere in the body, it is not fair to claim that the reduced transcription has anything to do with the arch expression domain or is a sign of reduced chondrogenesis. Sox9a levels can also be generally variable at this stage; it would thus be helpful to show multiple examples of the immunostaining result in a supplementary figure.

8. The authors assayed for osteogenic markers at 36 hpf, which is well before any definitive pre-osteoblasts can be detected in the pharyngeal arches, much less early or late osteoblasts. 48 hpf or later would be a better time point to assess *runx2a/b* expression and have some confidence that the expression was relevant; even later stages for the other markers. I am not convinced that the antibody staining truly reflects Runx2 expression. The osteogenic qPCR results suffer from the same problem as the rest of the qPCR data in this study: these genes are not specifically expressed only in the arches or only in one cell type, so assaying expression in whole embryos is not particularly informative. The conclusions made in the discussion about osteo/chondro progenitor dysregulation (lines 373-376) are not well supported.

9. While the alkaline phosphatase staining is likely real, activity of this enzyme is not limited to osteoblasts, and the structures appear to be mis-identified here. The staining in the gill arches looks more like gill filaments, especially as there isn't really any bone forming at 3 dpf in this region. There are certainly no sutures formed yet at 3 dpf – the calvarial bones don't even start to grow over the roof of the skull until about 2 wpf (Kanther et al. 2019; Parichy et al. 2009); whatever is stained on the top of the head must be something else.

10. The Fgf8 immunostaining results bear no real resemblance to the highly specific published *fgf8a* expression patterns (e.g. http://zfin.org/ZDB-IMAGE-020319-117), making me question the antibody fidelity.

11. The authors quantify *fgf8a* and *ncl* mRNA levels in controls and mutants (whole embryos again) at 4 stages and show that the relative levels of both decline in the mutants in a similar pattern. This is a puzzling way to show these data. The graph implies that *fgf8a* transcript levels approach zero in mutants vs. controls by 24 hpf. However, what do the non-normalized data look like? Are the levels in controls quite low already? Are the differences between wt and mutant consistent and significant? If their model is that Ncl binds and stabilizes *fgf8a* RNA, then wouldn't it make more sense for the divergence between controls and mutants to become notable when the protein is first lost, between 12 and 18 hpf (Fig. 1)? How were the embryos genotyped in this qPCR experiment? The increased affinity of Ncl for *fgf8a* vs. *actb* RNA is also not very persuasive – the two mRNAs are present at such different levels, and binding does not in any case necessarily indicate that Ncl is protecting *fgf8a* RNA from

© 2022. Published by The Company of Biologists under the terms of the Creative Commons Attribution License (https://creativecommons.org/licenses/by/4.0/).

degradation.

12. Rescuing what appears to be a commonplace "sick embryo" phenotype by exposure to FGF8 protein in culture does not necessarily mean that *fgf8a* is the critical dysregulated target affecting NC development; it could also be argued that FGF8 supplementation produced a general mitogenic effect that simply helped mutants bypass the wave of cell death normally experienced around 24 hpf.

13. Fig. 6D purports to show that FGF8 supplementation of *ncl* mutants does not require restoration of rRNA transcription, as mutants treated with FGF8 plus the BMH21 inhibitor that blocks RNA Polymerase I activity were rescued similar to the mutant treated with FGF8 alone. However, no mutant treated with BMH21 alone is shown for comparison, and the wild-type controls treated with FGF8+BMH21 are quite impaired – the authors do not explain this discrepancy.

14. The authors cannot fairly make the conclusion that FGF8 rescues skeletal development via stimulation of *bmp2* expression. The *bmp2* qPCR data is again from whole embryos, so may not be relevant to facial development at all. Also, to make this connection, the authors would need to show that the rescuing effects of FGF8 are negated when the BMP pathway is inhibited or when *bmp2* is knocked out. BMP2 supplementation could be independently rescuing the general sickly embryo phenotype in the same mitogenic way FGF8 might be. There is insufficient data here to put Ncl, Fgf8, and Bmp2 into a coherent pathway.

15. It would be helpful to comment whether or not any other model organism mutants have been made for this gene, or whether pathogenic variants have been documented in clinical populations.

**Additional minor comments**

1. Please clarify in the results section and the legend that the images shown in Fig. 1 are immunostains rather than in situ hybridizations.
2. Legend to Fig. 1D notes that Ncl has become confined to the nucleus – this is not discernible in the image; an inset should be provided if this statement is retained.
3. The images in Fig. 2/S1 purporting to show a midbrain-hindbrain defect are insufficiently magnified; the extent of the phenotype cannot be readily appreciated.
4. Note which regions of the embryo were included in the pHH3 and EdU quantification (Fig. S2).
5. The p53 morpholino results do not add any clarity and do not warrant inclusion.
6. Line 286: *fgf8a* is also expressed in the oral ectoderm.
7. Correct typos on line 292.

Reviewer 2

*Advance summary and potential significance to field*

In this paper, the authors show quite convincingly that nucleolin mutants (ncl-/-) display decreased ribosomal RNA synthesis, activation of the p53 pathway leading to apoptosis, and craniofacial deformities. The latter appear to be independent of p53 activation, while a clear decrease in Fgf8a expression is observed in the ncl-/- mutants, that correlates with an interaction of nucleolin with fgf8a RNA. Finally, exogenous human FGF8 rescues the observed phenotype. The data are in general very convincing, controls correctly executed and shown.

*Comments for the author*

The one issue that may need further consideration is the involvement of ribosomal synthesis in the craniofacial phenotype. The authors correctly state in the introduction that mutations in genes involved in ribosomal RNA expression and maturation often lead to craniofacial defects. However, they study the effect of nucleolin, which plays a role in rRNA processing, but also other processes. They show that the craniofacial defects they observe in the ncl-/- mutants are indeed caused by a deficient stabilization of fgf8a RNA pointing to a mechanism potentially independent of ribosome

© 2022. Published by The Company of Biologists under the terms of the Creative Commons Attribution License (https://creativecommons.org/licenses/by/4.0/).

synthesis. They then however show that the rescue by human FGF8 actually also rescues ribosomal synthesis. Finally, the most puzzling result is shown in Fig 6D, where blocking rRNA transcription with BMH21 leads to severe cartilage defects in wt (ncl+/+), even in the presence of FGF8, while the ncl-/- is still rescued by this double treatment. How is this possible in the absence of rRNA transcription? In my opinion, this relation between the nucleolin mutant phenotype and ribosomal synthesis requires at the least some discussion.

Minor issues
p31: legend to Fig. 1: "By 18hpf, the expression of Nucleolin in ncl+/+ embryos is confined to the nucleus"?
this may be true, but cannot be concluded from Fig. 1D FGF8 + BMH21 treatment of the wt?
p35: legend to Fig. 3: "(D) p53 protein levels are higher in ncl-/- mutants …" should be "(E) …."
line 292: " reglulate oth mesenchymal" should probably read "regulate both …."

<u>Reviewer 3</u>

*Advance summary and potential significance to field*

In their manuscript “Nucleolin loss-of-function leads to aberrant FGF signaling and craniofacial anomalies”
Dash and Trainor characterized ncl mutant zebrafish. They first characterize the expression of ncl demonstrating that it is maternally provided and expressed fairly ubiquitously, although enriched in some tissues such as the pharyngeal arches. They show that the craniofacial skeleton is malformed in ncl mutants. In ncl mutants, protein levels of p53 are transiently elevated and there is reduced immunoprecipitation of p53 with Mdm2. The elevation in p53 results in elevated apoptosis but only some phenotypes are rescued in ncl;p53 double mutants. They find that migration into the pharyngeal arches appears normal in ncl mutants, but that chondrogenesis and osteogenesis are disrupted, with Sox9a being downregulated and Runx2 being upregulated. This finding lead to the Fgf pathway as a potential mechanism. They show that Ncl binds fgf8a mRNA and, in the absence of ncl, the levels of fgf8a mRNA and protein are reduced. The addition of exogenous Fgf8 partially restores craniofacial structures and elevates the levels of rRNA transcripts. The rescue occurs even when Pol I is chemically inhibited suggesting that the mechanism is independent of the role of Ncl in rRNA biogenesis. They go on to demonstrate that Fgf8 stimulation elevates the levels of bmp2 and that Bmp treatment can rescue ncl mutant embryos, suggesting this as the mechanism. The demonstration of non-rRNA functions for Ncl will be of broad interest to developmental biologists and, thus, warrants publication in Development.

*Comments for the author*

Concerns that should be dealt with prior to publication:
1). In their characterization of the ncl mutant phenotype, the authors state that development of medial ethmoid cells may be disrupted based on Alcian staining. This is difficult to ascertain in the images provided. Higher magnification images are needed.
2). Also related to Fig. 2, the authors demonstrate that the length of the trabeculae is reduced. Is this effect specific to the trabeculae? Is the ethmoid plate also reduced? Also, the focus of the manuscript is on the neural crest-derived skeleton, which is fine, but is the mesoderm-derived posterior neurocranium also reduced?
3). The statement that the neurocranium is not rescued in ncl;p53 double mutants is not well-supported by Fig. S3. In Fig. 2 the authors show that the trabeculae are shortened in ncl mutants. The authors should measure the trabeculae (and other measures that may come out of my 2nd concern) in the doubles to determine if there is rescue of the neurocranium. To this reviewer’s eye, the neurocranium does appear rescued. The image shown isn’t stained as darkly, but this isn’t a phenotype present in the single mutant and could simply be a difference in the prep. Additionally, including ncl-/-;p53+/+ siblings in this figure is needed to ensure that there isn’t a background effect in the double.
4). In a related concern, the morpholino data are highly concerning. The p53 morpholino-injected ncl mutants are much more disrupted than the double mutants. Even the control morpholino-injected mutants appear much worse than the ncl single mutants in Fig. 2J. Perhaps the ncl mutants are merely more sensitive to off target morpholino defects?

© 2022. Published by The Company of Biologists under the terms of the Creative Commons Attribution License (https://creativecommons.org/licenses/by/4.0/).

5) In general, the craniofacial phenotypes are not quantified. This makes it very difficult to ascertain the overall level of rescue/interaction in their various experiments. The authors should add quantification of these effects. Even simple measures like percentage of embryos with a basihyal and average number of ceratobranchials/teeth would be very helpful.
Minor concerns:
1). In the legend for Fig. 1 the authors state “Nucleolin expression is specific to the jaw of the embryo.”
Some different wording should be used because it does appear to be expressed elsewhere. Even within the head it is not limited to the jaw.
2). In the legend for Fig. 3 panel E is not referenced. There appears to be a typo, labeling it as D.
3). In Fig. 3, it is not clear that apoptosis is restricted to the MHB. Separating the red and blue channels would help the reader assess this claim.
4). Line 292, “oth” should be “other”.
5) In the methods the authors state they used the Walker and Kimmel bone and cartilage labeling protocol but then reference Kimmel 1995. Neither reference is in the reference list.
6) In many figures it is difficult to tell where there are significant differences between groups. A supplemental table listing the p values for all comparisons would be helpful.

---

**First revision**

<u>Author response to reviewers' comments</u>

**Response to Reviewers:**

We sincerely thank the reviewers for their thorough and insightful critiques of our paper, and their recognition of the importance of our work. We have revised our manuscript according to the comments and suggestions from the reviewers as described below. Changes to the text are highlighted for ease of reference. We trust the reviewers will now find the work satisfactorily addresses their concerns and is suitable for publication.

Reviewer 1 Advance Summary and Potential Significance to Field...

A recurring phenomenon in neural crest (NC) biology is that NC cells are disproportionately impaired by mutation of genes presumed to be of ubiquitous expression and function, e.g., those involved in rRNA transcription and ribosome biogenesis. Numerous hypotheses have been put forward to explain this curiosity, and it has been of interest to the Trainor lab for some time. In this manuscript, Dash & Trainor add nucleolin to this list of ubiquitous genes that NC cells are hypersensitive to the loss of. Ncl is a phosphoprotein that is a major component of the nucleolus (but also expressed in the cytoplasm) and has documented roles in rRNA processing and mRNA/DNA binding. Using an existing zebrafish insertional mutant allele for ncl, the authors show a clear downregulation of rRNA transcription and increased levels of p53 protein/apoptosis in mutant embryos, as well mild craniofacial malformations and lethality. They are able to rescue most of these phenotypes by mutating p53 or by treating with recombinant FGF8 or BMP2, supporting the conclusion that the ncl mutant is suffering from a mild neurocristopathy.

Reviewer 1 Comments for the Author:
Major comments
1. The ncl mutant craniofacial phenotype closely resembles the common “sickly” facial development phenotype associated with generally impaired development or morpholino-associated toxicity (hypoplastic cartilages, inverted ceratohyal, poorly stained and perpendicular ceratobranchials, small eyes, misshapen head, uninflated swim bladder). It is not surprising that a mild neurocristopathy caused by mutation in a housekeeping gene like ncl would take this form, or that it would be so readily rescued by treatments that counteract cell death. The authors should place this overall phenotype in the context of others in the literature.

- We first want to clarify that no morpholinos were used to generate *ncl* mutants and the phenotype is not associated with toxicity from morpholino injection. While we understand

© 2022. Published by The Company of Biologists under the terms of the Creative Commons Attribution License (https://creativecommons.org/licenses/by/4.0/). 

that a general "sickly" phenotype has been observed in other zebrafish mutants, the phenotype for *ncl*$^{-/-}$ mutant zebrafish has not been described previously. Moreover, each of these phenotypes are unique in some respects. For example, *polr1c* and *polr1d* mutants have a similar ceratohyal and ceratobranchial phenotypes as *ncl*$^{-/-}$ mutants, however their basihyal is intact while it is missing in *ncl*$^{-/-}$ mutants (Watt et al., 2016, PLOS Genetics). We have however addressed this issue in the discussion as suggested by the reviewer. We also note that similar phenotypes have been previously observed and that a follow up of such phenotypes will be analyzed in the future.

2. The authors observe that Ncl protein is maternally supplied, ubiquitously expressed (Fig. 1), and not significantly depleted in mutants until after 12 hpf. Transcript levels in mutants are likewise similar to controls at 18 hpf and only appear to decline (to ~50% of control level) after this stage (Fig. 5B). This issue of maternal loading and the timing of decline warrants more attention. As the zygotic mutants are not adult-viable, I recognize that it is not particularly simple to create a maternal-zygotic mutant to determine how essential this maternal contribution is. However, whether the NCCs are specifically impacted because an earlier/broader requirement for the gene is rescued by the maternal contribution could be discussed in more detail.

- This is an important point and accordingly we have added a paragraph in the discussion to include maternal Nucleolin contribution to the broader requirement of Nucleolin in early development.

3. It is unclear when the rRNA qPCR experiment shown in Fig. 3A was performed, and whether the effect changes over time. If it is prominent early and then normalized through an unknown mechanism, that would support (in part) the authors' model that Nucleolin's critical time of function is in the early pharyngula stages.

- We apologize for the omission of the stage at which qPCR was performed for rRNA synthesis. We have now added this to our result section. We have also added a qPCR (Figure S2A) at three other stages for comparison and show that rRNA synthesis is generally reduced in *ncl*$^{-/-}$ mutants starting at 18 hpf. rRNA levels in *ncl*$^{-/-}$ mutants do not recover until 36 hpf. We hypothesize that Nucleolin is necessary for early developmental stages based on our soon to be published data in mouse embryos. However, as the reviewer mentioned in their earlier point it is not particularly simple to create a maternal-zygotic zebrafish mutant. Nucleolin's function in early embryonic development will however be explored further in our mouse studies.

4. Ncl has previously been shown to modify p53 levels through both direct and indirect mechanisms. Mutants show unaltered p53 transcript levels but a peak in protein accumulation at the stage (~24 hpf) when cell death is elevated. The authors show that Ncl does not bind p53 mRNA in this context but does interact with the protein in wild-type embryos (stage not noted). However, because p53 protein levels are transiently increased in mutants, this result would argue against Ncl contributing to p53 stability.

- The very fact that p53 expression is increased transiently suggests that it gets degraded soon after being translated and its stability is reduced. p53 protein has a short half-life and without the stabilizing influence of Nucleolin, it is downregulated instead of being continuously maintained. Therefore, we conclude that Nucleolin binding to p53 must contribute to its stability as has been described previously (Saxena et al., 2006; Takagi et al., 2005).

The author then did a rather confusing experiment wherein they performed immunoprecipitation for the p53 ubiquitinator Mdm2 and then blotted simultaneously for Mdm2 and p53, in the same color. The blotting result is interpreted as supposedly decreased pulldown of both Mdm2/p53 in mutants, supposedly meaning that their interaction level is lower, which in turn could explain why p53 levels are transiently higher. This conclusion is problematic on multiple levels – the IP blot in Fig. 2H is smeared and impossible to interpret; less Mdm2 on the mutant blot (if true) would imply less Mdm2 protein pulled down, not less Mdm2-p53 interaction.

- We have now repeated our immunoprecipitation experiment and shown separate blots for p53 and Mdm2 to show clear bands (Fig. 3H). Both p53 and Mdm2 bands could only be detected at the 680 wavelength and therefore, the blot had to be probed for both p53 and Mdm2 in the same channel. Further, the two proteins are fairly close together in size and

© 2022. Published by The Company of Biologists under the terms of the Creative Commons Attribution License (https://creativecommons.org/licenses/by/4.0/).

therefore, we divided our immunoprecipitate into two separate gels and blots and immunoprobed for p53 and Mdm2 separately. Since we performed immunoprecipitation with p53 and observe less Mdm2 pulldown in *ncl*$^{-/-}$ mutants compared to controls, we conclude that Mdm2-p53 interaction is reduced in *ncl*$^{-/-}$ mutants.

It is not clear whether p53 protein levels are still elevated at 28 hpf, when the IP was done.

- Our input lane in Fig. 3H shows higher p53 protein expression in *ncl*$^{-/-}$ mutants compared to controls. We further observe high general apoptosis at 28hpf in the *ncl*$^{-/-}$ mutants corroborating our western data (Fig. 6B).

The authors' conclusions that "the initial accumulation of p53 is a result of reduced interactions between Mdm2 and p53" and "The subsidence of p53 activity at later stages might therefore be due to a lack of Nucleolin stabilization of p53 protein" do not seem fully justified by the data presented.

- Since we cannot perform protein stability assays in vivo in zebrafish, we agree that our conclusion cannot be justified just by the data presented. However, we reference previous literature (Saxena et al., 2006; Takagi et al., 2005) in concert with our temporal data to draw such conclusions.

5. The p53 rescue looks quite robust, though the authors should include a picture of an ncl-/-; p53+/+ larva in Fig. S3A to show that the single mutant phenotype is the same as reported in the main text on this genetic background. Also, weak Alcian staining of ceratohyal chondrocytes is a fairly common staining artifact and should be interpreted with caution. It goes too far to say that these dKOs "die around 10-12 dpf due to these cranioskeletal defects" and that "the skeletal defects in ncl-/- mutant zebrafish are not p53 dependent" – they are quite well rescued!

- We have now included *ncl*$^{-/-}$*;p53*$^{+/+}$ in Figure S4. We agree that weak alcian staining could be interpreted as staining artifact. However, we have performed this analysis in five different clutches and observed similarly lower levels of alcian staining in the ceratobranchials and in the viscerocranium in *ncl*$^{-/-}$*;p53*$^{-/-}$ embryos (n = 82). We are cautious in not discounting this data and prefer to not label *ncl*$^{-/-}$*;p53*$^{-/-}$ embryos as rescued, especially compared to our own rescue data with Fgf8 supplementation. Further, when we compare it to *p53*$^{-/-}$ rescue in other zebrafish mutants such as *polr1c* and *polr1d* mutants (Watt et al., 2016, PLOS Genetics), we find that while *polr1c*$^{-/-}$*;p53*$^{-/-}$ and *polr1d*$^{-/-}$*;p53*$^{-/-}$ has rescued certahyal polarity and ceratobranchial chondrogenesis, we do not observe any rescue of certabranchial chondrogenesis in *ncl*$^{-/-}$*;p53*$^{-/-}$ mutants.

6. sox10 mRNA is expressed in migrating NCCs and non-ectomesenchymal lineages but is rapidly downregulated ~18 hpf when ectomesenchyme-fated NCCs arrive in the pharyngeal region. It is then reexpressed in differentiating chondrocytes after ~56 hpf. I am thus suspicious of the hazy immunostaining in the arches at 36 hpf (Fig. S4D) – the punctate nuclear staining elsewhere in the body looks real but the arch staining does not.

- While the staining in the arch does look hazy compared to the bright nuclear staining elsewhere in the zebrafish embryo, this staining is nuclear and specific to the arch cells. It is possible that Sox10 protein has a longer half-life than *sox10* mRNA. We have also observed *sox10* mRNA expression in the pharyngeal arches at 36 hpf in other published literature (Chen et al., 2018, FEBS Journal; Meulen et al., 2020, Front. Cell Dev. Biol.). However, we have removed specific mention of pharyngeal arch in our description of the results.

7. The authors present qPCR data showing that sox9a and col2a1a are downregulated in mutants [at an undefined stage] and that Sox9a protein levels look slightly lower in the posterior arches at 36 hpf. However, because they appear to have used whole embryos for the RNA extraction/qPCR experiment and because these genes are expressed elsewhere in the body, it is not fair to claim that the reduced transcription has anything to do with the arch expression domain or is a sign of reduced chondrogenesis. Sox9a levels can also be generally variable at this stage; it would thus be helpful to show multiple examples of the immunostaining result in a supplementary figure.

- Given that the *ncl* mutation was germline and not conditional in NCC, we tested for expression of chondrogenic and osteogenic markers in the whole embryo with the exception

© 2022. Published by The Company of Biologists under the terms of the Creative Commons Attribution License (https://creativecommons.org/licenses/by/4.0/).

of the tip of the tail. We do observe a general downregulation of Sox9a protein in our immunostaining. Since the major function of *Sox9a* is to enable chondrogenesis as evidenced by the *sox9a*$^{-/-}$ mutation (Flanagan-Steet et al., 2016, J Bone Miner Res), we feel comfortable in our conclusions that reduced Sox9a expression contributes to reduced chondrogenesis.

- We respect that the reviewer has observed variable Sox9a levels in zebrafish at 36 hpf, however, we observe consistent staining, and we provide examples below. We do not think it is necessary for this data to be included as a supplementary figure because we are going to include our replicate data in our Institutional data repository.

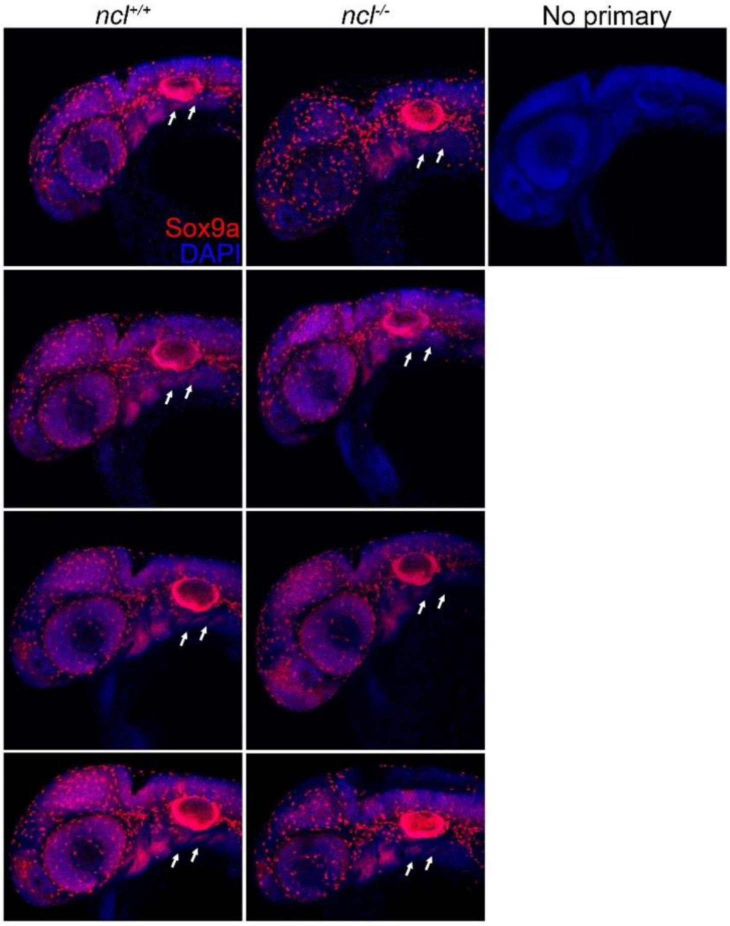


8. The authors assayed for osteogenic markers at 36 hpf, which is well before any definitive pre-osteoblasts can be detected in the pharyngeal arches, much less early or late osteoblasts. 48 hpf or later would be a better time point to assess runx2a/b expression and have some confidence that the expression was relevant; even later stages for the other markers. I am not convinced that the antibody staining truly reflects Runx2 expression. The osteogenic qPCR results suffer from the same problem as the rest of the qPCR data in this study: these genes are not specifically expressed only in the arches or only in one cell type, so assaying expression in whole embryos is not particularly informative. The conclusions made in the discussion about osteo/chondro progenitor dysregulation (lines 373-376) are not well supported.

- We have now added Runx2 staining at 3dpf in Figure 4 and show that Runx2 is overexpressed in *ncl*$^{-/-}$ mutants compared to *ncl*$^{+/+}$ embryos that concurs with our increased Runx2 expression at 36 hpf. Further, our Runx2 protein expression at both 36 hpf and 3dpf follows the published expression pattern of *runx2a* and runx2b mRNA expression pattern (Li et al., 2009, Patterns & Phenotypes). While we do agree with the reviewer that pre-osteoblasts are not detected in the pharyngeal arches at 36 hpf, osteochondroprogenitors at this stage do express Runx2 (Li et al., 2009, Patterns & Phenotypes). To further

© 2022. Published by The Company of Biologists under the terms of the Creative Commons Attribution License (https://creativecommons.org/licenses/by/4.0/).

corroborate our staining pattern, we provide our no primary control in the figure below, which shows the fidelity of our antibody.

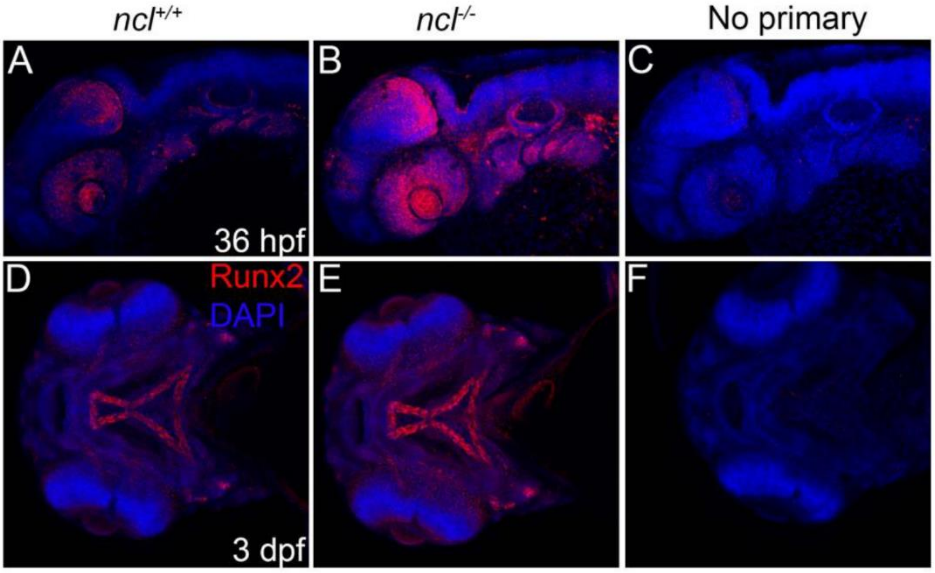


- We further performed alkaline phosphatase staining at stages 3dpf and 5dpf to specifically label osteoblasts and observe a significant downregulation of alkaline phosphatase positive cells in the ceratobranchials in *ncl*$^{-/-}$ mutants.

- We agree that our qPCR data was performed using the entire embryo and not with just the arch tissue, which could skew the data. However, all our qPCR data is followed up with further experiments. For example, *sox9a* qPCR is followed up with *col2a1* qPCR, Sox9a protein expression, alcian blue staining at 3dpf, 5dpf, 8dpf and 10dpf and hypoplastic cartilage phenotype. *runx2a* and *runx2b* qPCR is followed up by *cola12*, *col10a1*, *sp7*, *spp1* and *bglap* qPCR, Runx2 protein expression at 36 hpf and 3dpf, alkaline phosphatase staining at 3dpf and 5dpf as well as the alizarin staining at 5dpf, 8dpf and 10dpf as well as the hypoplastic bone phenotype. In addition, previous literature suggests that *col1a2* (Thisse, C., and Thisse, B. (2005) High Throughput Expression Analysis of ZF-Models Consortium Clones. ZFIN Direct Data Submission (http://zfin.org), *col10a1a* (Li et al, 2009, Dev. Dyn) and *sp7* (Thisse, C., and Thisse, B. (2005) High Throughput Expression Analysis of ZF-Models Consortium Clones. ZFIN Direct Data Submission (http://zfin.org) and *spp1* (Torregroza et al., 2012, PLOS One) are expressed in the craniofacial tissue at 36 hpf. Based on all of the above-mentioned data, we feel that our conclusions are well supported.

9. While the alkaline phosphatase staining is likely real, activity of this enzyme is not limited to osteoblasts, and the structures appear to be mis-identified here. The staining in the gill arches looks more like gill filaments, especially as there isn't really any bone forming at 3 dpf in this region. There are certainly no sutures formed yet at 3 dpf – the calvarial bones don't even start to grow over the roof of the skull until about 2 wpf (Kanther et al. 2019; Parichy et al. 2009); whatever is stained on the top of the head must be something else.

- We agree that calvarial bones are present only at 2wpf. The staining at the top of the head labels blood vessels (Kamei et al.,2010, Methods Cell Biol). We have now removed this data from the paper.

10. The Fgf8 immunostaining results bear no real resemblance to the highly specific published fgf8a expression patterns (e.g. http://zfin.org/ZDB-IMAGE-020319-117), making me question the antibody fidelity.

- Since Fgf8 is a secreted protein, its mRNA expression pattern is not identical to its protein localization. A similar difference in mRNA and protein distribution is observed in mouse embryos (https://www-ncbi-nlm-nih-gov/pmc/articles/PMC3913879/ Figure 4). In addition, the antibody was tested for fidelity by Genetex

© 2022. Published by The Company of Biologists under the terms of the Creative Commons Attribution License (https://creativecommons.org/licenses/by/4.0/).

(https://www.genetex.com/Product/Detail/Fgf8a-antibody/GTX128126#datasheet) and has been used in Giffen et al., 2019, Front. NeuroSci. In addition, we provide our no primary control embryo staining in the figure below. Therefore, we think it is true expression pattern.

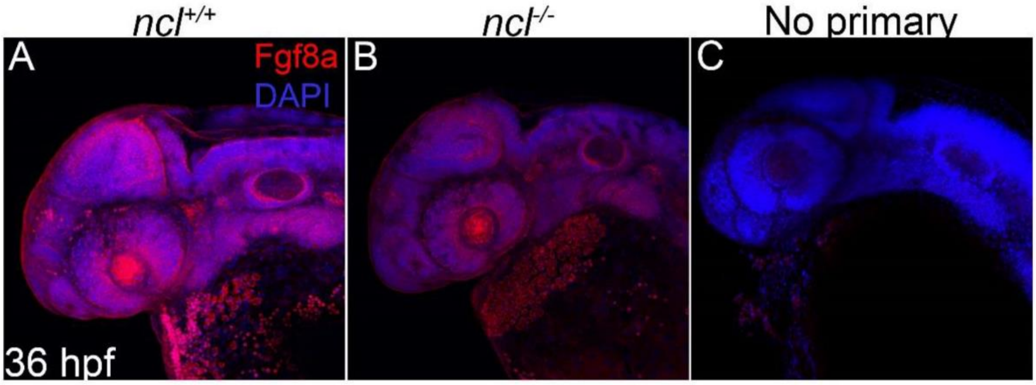


11. The authors quantify fgf8a and ncl mRNA levels in controls and mutants (whole embryos again) at 4 stages and show that the relative levels of both decline in the mutants in a similar pattern. This is a puzzling way to show these data. The graph implies that fgf8a transcript levels approach zero in mutants vs. controls by 24 hpf. However, what do the non-normalized data look like? Are the levels in controls quite low already?

- We have now added the non-normalized data for *fgf8a* and *ncl* mRNA expression in Figure S2 along with rRNA, *p53* and *p21* mRNA in *ncl*$^{+/+}$ and *ncl*$^{-/-}$ embryos. The mRNA expression levels in the controls are similar between these developmental stages.

Are the differences between wt and mutant consistent and significant?

- Yes, the differences between *ncl*$^{+/+}$ and *ncl*$^{-/-}$ embryos are consistent and significant (Figure S2).

If their model is that Ncl binds and stabilizes fgf8a RNA, then wouldn't it make more sense for the divergence between controls and mutants to become notable when the protein is first lost, between 12 and 18 hpf (Fig. 1)?

- We did not assay for *fgf8a* mRNA at stages prior to 18hpf in *ncl*$^{-/-}$ embryos due to difficulty in differentiating mutants from controls prior to performing RNA isolation and qPCR experiment. Phenotypically, *ncl*$^{-/-}$ embryos are indistinguishable from *ncl*$^{+/+}$ and *ncl*$^{+/-}$ embryos prior to 24 hpf. In addition, prior to 18hpf there is no tail bud that can be separated from the embryo to perform genotyping. Therefore, we performed qPCR experiments beginning at 18hpf and we observe a downregulation of *fgf8a* at 24 hpf. In addition, *fgf8a* downregulation at early developmental stages results in agenesis of the cerebellum and MHB organizer (Reifers et al., 1998). Since we do not observe such drastic phenotypes in *ncl*$^{-/-}$ phenotypes, this further corroborates our observations data of *fgf8a* downregulation beginning at 24 hpf.

- While we agree that *fgf8a* mRNA downregulation at 18hpf would be a simpler mechanism, the quantity of mRNA present in a cell is determined by a balance between transcription and degradation (Hasan et al., 2014, PLOS Genetics). Since, we do not yet have an assay to perform mRNA degradation kinetics experiments on zebrafish, we are unable to answer this question in this manuscript.

How were the embryos genotyped in this qPCR experiment?

- The embryos were genotyped individually using the tip of the tail tissue (tissue right of the red line in the figure below). After identification of mutants and wildtypes from a clutch, 10 embryos of the same genotype were pooled together for RNA isolation. This has been now added to the methods and materials section.

© 2022. Published by The Company of Biologists under the terms of the Creative Commons Attribution License (https://creativecommons.org/licenses/by/4.0/).

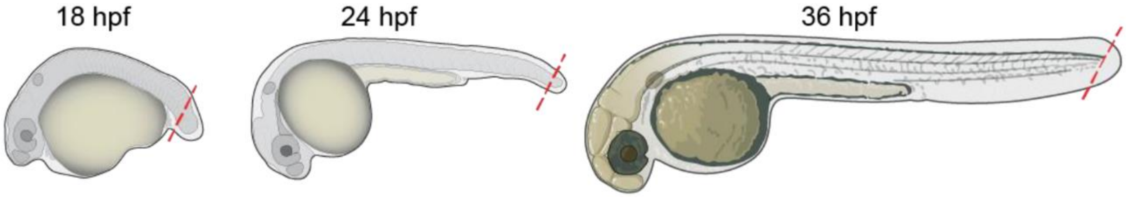


The increased affinity of Ncl for fgf8a vs. actb RNA is also not very persuasive – the two mRNAs are present at such different levels, and binding does not in any case necessarily indicate that Ncl is protecting fgf8a RNA from degradation.

- We agree that the level of expression of *fgf8a* and *actb* are very different from each other. *actb* is expressed at least 2-fold higher than *fgf8a* based on qPCR data. Therefore, observing that Nucleolin antibody does not bind to such an abundant RNA molecule that is readily available suggests Nucleolin binding to fgf8a mRNA is specific. Further, we determined the binding locus of Nucleolin from previous literature and bioinformatically determined it to be present in the 5'UTR of the *fgf8a* mRNA. An RNA binding protein binding to a 5'UTR of a mRNA has two probable functions 1) it can stabilize/degrade mRNA or 2) promote/hinder protein translation. Combined with our data that *fgf8a* mRNA reduces over time in the *ncl*$^{-/-}$ mutants, this suggests that mRNA is affected, which results in reduced translation, indicating that Nucleolin must stabilize the *fgf8a* mRNA. We have now added further explanation of the experiment and our conclusions in the result and discussion section.

12. Rescuing what appears to be a commonplace "sick embryo" phenotype by exposure to FGF8 protein in culture does not necessarily mean that fgf8a is the critical dysregulated target affecting NC development; it could also be argued that FGF8 supplementation produced a general mitogenic effect that simply helped mutants bypass the wave of cell death normally experienced around 24 hpf.

- We agree that FGF8 supplementation leads to mitogenic effect that bypasses the wave of initial cell death as observed in Fig 6B. However, the initial cell death observed at 24 hpf does not prevent neural crest cell migration to the branchial arches in *ncl*$^{-/-}$ mutants without FGF8 supplementation (Fig. S5A). The volume of the arch and the fluorescence intensity of *sox10-egfp* at 36 hpf *ncl*$^{+/+}$ and *ncl*$^{-/-}$ embryos are comparable. In addition, *ncl*$^{-/-}$ embryos have higher proliferation rates compared to *ncl*$^{+/+}$ embryos prior to any drug treatments (Fig. S3). Cell death in *ncl*$^{-/-}$ embryos at 36 hpf is dorsal and does not affect branchial arches (Fig. 3M-N'). Therefore, we hypothesize that the hypoplastic cartilage and bone phenotype of the *ncl*$^{-/-}$ mutants is due to a defect in differentiation of neural crest cells and FGF8 supplementation rescues this defect. To test this hypothesis, we treated the *ncl*$^{+/+}$ and *ncl*$^{-/-}$ embryos with FGF8 at 30 hpf when the cell death is significantly reduced. We observe a similar rescue of the cartilage and bone phenotype in *ncl*$^{-/-}$ embryos when treated with FGF8 at 18 and 30 hpf (Fig. S8), suggesting that FGF8 treatment regulates chondrogenesis and osteogenesis.

13. Fig. 6D purports to show that FGF8 supplementation of ncl mutants does not require restoration of rRNA transcription, as mutants treated with FGF8 plus the BMH21 inhibitor that blocks RNA Polymerase I activity were rescued similar to the mutant treated with FGF8 alone. However, no mutant treated with BMH21 alone is shown for comparison, and the wild-type controls treated with FGF8+BMH21 are quite impaired – the authors do not explain this discrepancy.

- We have now added a new panel to Fig 6D with just BMH21 treatment. We observe a cartilage hypoplasia phenotype in *ncl*$^{+/+}$ larvae severe than the *ncl*$^{-/-}$ phenotype, possibly because of p53 response to ribosomal stress in *ncl*$^{+/+}$ larvae. However, *ncl*$^{-/-}$ embryos do not have a sustained expression of p53 to cause general apoptosis and loss of chondrogenesis. We hypothesize a similar mechanism for the FGF8+BMH21 treated embryos, where the *ncl*$^{+/+}$ embryos have a p53 response that FGF8 supplementation cannot override, while *ncl*$^{-/-}$ embryos do not exhibit p53-dependent general apoptosis after 30hpf (Fig. S9).

© 2022. Published by The Company of Biologists under the terms of the Creative Commons Attribution License (https://creativecommons.org/licenses/by/4.0/). 

14. The authors cannot fairly make the conclusion that FGF8 rescues skeletal development via stimulation of bmp2 expression. The bmp2 qPCR data is again from whole embryos, so may not be relevant to facial development at all. Also, to make this connection, the authors would need to show that the rescuing effects of FGF8 are negated when the BMP pathway is inhibited or when bmp2 is knocked out. BMP2 supplementation could be independently rescuing the general sickly embryo phenotype in the same mitogenic way FGF8 might be. There is insufficient data here to put Ncl, Fgf8, and Bmp2 into a coherent pathway.

- Experimentally, we are unable to test this hypothesis since there are no Bmp2 specific inhibitors. Noggin, which is a Bmp inhibitor inhibits all Bmp pathways, which will lead to off target effects. Additionally, morpholino downregulation of Bmp2 will result in downregulation of Bmp2 at one cell stage, which is far earlier than the downregulation in ncl mutants, leading to defects irrespective of Nucleolin deficiency. However, as we described in point 12, the mitogenic pathway alone cannot explain the rescue either. Given that *ncl*$^{-/-}$ mutants exhibit *bmp2* downregulation, which is rescued by FGF8 supplementation suggests that FGF8 treatment upregulates *bmp2* expression. BMP2 treatment further rescues the craniofacial phenotype of *ncl*$^{-/-}$ mutants. We feel justified in suggesting that FGF8 regulates Bmp2 expression and both of these pathways rescue *ncl*$^{-/-}$ phenotypes.

15. It would be helpful to comment whether or not any other model organism mutants have been made for this gene, or whether pathogenic variants have been documented in clinical populations.

- Nucleolin has been downregulated using morpholinos in Xenopus, where the craniofacial cartilage phenotype is similar to the *ncl*$^{-/-}$ zebrafish mutant (Delhermite et al., 2022, PLOS Genetics). We have now added this to our discussion. No clinical pathogenic variants have been described to date.

Additional minor comments

1. Please clarify in the results section and the legend that the images shown in Fig. 1 are immunostains rather than in situ hybridizations.
   - We have now clarified this in the result and legend section.
2. Legend to Fig. 1D notes that Ncl has become confined to the nucleus – this is not discernible in the image; an inset should be provided if this statement is retained.
   - We have now added a section of 18hpf WT embryo in Fig 1D" to show nuclear staining of Nucleolin.

3. The images in Fig. 2/S1 purporting to show a midbrain-hindbrain defect are insufficiently magnified; the extent of the phenotype cannot be readily appreciated.
   - We have now added higher magnification images of the Midbrain-hindbrain boundary in Fig. 2C' and D'.

4. Note which regions of the embryo were included in the pHH3 and EdU quantification (Fig. S2).
   - The whole head from frontonasal prominence to the pharyngeal arches were used to quantify pHH3 and EdU and has now been clarified in both result section and the figure legends.

5. The p53 morpholino results do not add any clarity and do not warrant inclusion.
   - We agree with the reviewer and have removed the data from the paper.

6. Line 286: fgf8a is also expressed in the oral ectoderm.
   - We could not confirm fgf8a expression in the oral ectoderm in zebrafish from published literature. We acknowledge the expression of Fgf8 in the oral ectoderm in both mice and chick (Compagnucci et al., 2013, Dev Biol). We will gladly add oral ectoderm to our list of tissues where fgf8a is expressed if the reviewer can point us to a paper or provide us unpublished data.

7. Correct typos on line 292.
   - We have now corrected the typo on line 292.

© 2022. Published by The Company of Biologists under the terms of the Creative Commons Attribution License (https://creativecommons.org/licenses/by/4.0/).

Reviewer 2 Advance Summary and Potential Significance to Field:

In this paper, the authors show quite convincingly that nucleolin mutants (ncl-/-) display decreased ribosomal RNA synthesis, activation of the p53 pathway leading to apoptosis, and craniofacial deformities. The latter appear to be independent of p53 activation, while a clear decrease in Fgf8a expression is observed in the ncl-/- mutants, that correlates with an interaction of nucleolin with fgf8a RNA. Finally, exogenous human FGF8 rescues the observed phenotype. The data are in general very convincing, controls correctly executed and shown.

Reviewer 2 Comments for the Author:

The one issue that may need further consideration is the involvement of ribosomal synthesis in the craniofacial phenotype. The authors correctly state in the introduction that mutations in genes involved in ribosomal RNA expression and maturation often lead to craniofacial defects. However, they study the effect of nucleolin, which plays a role in rRNA processing, but also other processes. They show that the craniofacial defects they observe in the ncl-/- mutants are indeed caused by a deficient stabilization of fgf8a RNA, pointing to a mechanism potentially independent of ribosome synthesis. They then however show that the rescue by human FGF8 actually also rescues ribosomal synthesis. Finally, the most puzzling result is shown in Fig 6D, where blocking rRNA transcription with BMH21 leads to severe cartilage defects in wt (ncl+/+), even in the presence of FGF8, while the ncl-/- is still rescued by this double treatment. How is this possible in the absence of rRNA transcription? In my opinion, this relation between the nucleolin mutant phenotype and ribosomal synthesis requires at the least some discussion.

- We have now added a new panel to Fig 6D with just BMH21 treatment in *ncl*$^{+/+}$ and *ncl*$^{-/-}$ embryos. We observe a cartilage hypoplasia phenotype in *ncl*$^{+/+}$ larvae that is more severe than the *ncl*$^{-/-}$ phenotype, possibly because of p53 response to ribosomal stress in *ncl*$^{+/+}$ larvae. However, *ncl*$^{-/-}$ embryos do not have a sustained expression of p53 to cause general apoptosis and loss of chondrogenesis, because p53 stablilization is short lived in the absence of Nucleolin. We hypothesize a similar mechanism for the FGF8+BMH21 treated embryos, where the *ncl*$^{+/+}$ embryos have a p53 response that FGF8 supplementation cannot override, while *ncl*$^{-/-}$ embryos do not exhibit p53-dependent general apoptosis after 30hpf (Fig. S9). This is now added to our discussion.

Minor issues:
p31: legend to Fig. 1: "By 18hpf, the expression of Nucleolin in ncl+/+ embryos is confined to the nucleus"? this may be true, but cannot be concluded from Fig. 1D

- We have now added a section of 18hpf WT embryo in Fig 1D” to show nuclear staining of Nucleolin.

FGF8 + BMH21 treatment of the wt?
p35: legend to Fig. 3: "(D) p53 protein levels are higher in ncl-/- mutants …" should be "(E) …."

- We thank the reviewer for pointing out our typographical error. This has been corrected.

line 292: " reglulate oth mesenchymal" should probably read "regulate both …."

- We again thank the reviewer for pointing out our typographical error. We have corrected it to state “regulate other”.

Reviewer 3 Advance Summary and Potential Significance to Field:

In their manuscript “Nucleolin loss-of-function leads to aberrant FGF signaling and craniofacial anomalies”
Dash and Trainor characterized ncl mutant zebrafish. They first characterize the expression of ncl, demonstrating that it is maternally provided and expressed fairly ubiquitously, although enriched in some tissues such as the pharyngeal arches. They show that the craniofacial skeleton is malformed in ncl mutants. In ncl mutants, protein levels of p53 are transiently elevated and there is reduced immunoprecipitation of p53 with Mdm2. The elevation in p53 results in elevated apoptosis but only some phenotypes are rescued in ncl;p53 double mutants. They find that migration into the pharyngeal arches appears normal in ncl mutants, but that chondrogenesis and osteogenesis are disrupted, with Sox9a being downregulated and Runx2 being upregulated. This finding lead to the

© 2022. Published by The Company of Biologists under the terms of the Creative Commons Attribution License (https://creativecommons.org/licenses/by/4.0/). 

Fgf pathway as a potential mechanism. They show that Ncl binds fgf8a mRNA and, in the absence of ncl, the levels of fgf8a mRNA and protein are reduced. The addition of exogenous Fgf8 partially restores craniofacial structures and elevates the levels of rRNA transcripts. The rescue occurs even when Pol I is chemically inhibited, suggesting that the mechanism is independent of the role of Ncl in rRNA biogenesis. They go on to demonstrate that Fgf8 stimulation elevates the levels of bmp2 and that Bmp treatment can rescue ncl mutant embryos, suggesting this as the mechanism. The demonstration of non-rRNA functions for Ncl will be of broad interest to developmental biologists and, thus, warrants publication in Development.

Reviewer 3 Comments for the Author:

Concerns that should be dealt with prior to publication:

1). In their characterization of the ncl mutant phenotype, the authors state that development of medial ethmoid cells may be disrupted based on Alcian staining. This is difficult to ascertain in the images provided. Higher magnification images are needed.

- We have now added higher magnification images of the alcian blue staining in Figure 2 K and L, which show the absence of medial ethmoid cells.

2). Also related to Fig. 2, the authors demonstrate that the length of the trabeculae is reduced. Is this effect specific to the trabeculae? Is the ethmoid plate also reduced? Also, the focus of the manuscript is on the neural crest-derived skeleton, which is fine, but is the mesoderm-derived posterior neurocranium also reduced?

- We measured the length of the ethmoid plate of both *ncl*$^{+/+}$ and *ncl*$^{-/-}$ larvae and found that the size of the ethmoid plate in *ncl*$^{-/-}$ larvae is similar to the *ncl*$^{+/+}$ larvae as shown in the figure on the right. The posterior neurocranium is also not significantly different.

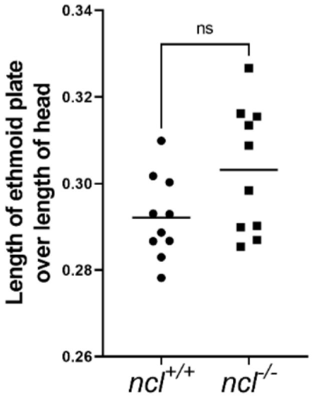


3). The statement that the neurocranium is not rescued in ncl;p53 double mutants is not well-supported by Fig. S3. In Fig. 2 the authors show that the trabeculae are shortened in ncl mutants. The authors should measure the trabeculae (and other measures that may come out of my 2nd concern) in the doubles to determine if there is rescue of the neurocranium. To this reviewer's eye, the neurocranium does appear rescued. The image shown isn't stained as darkly, but this isn't a phenotype present in the single mutant and could simply be a difference in the prep. Additionally, including ncl-/-;p53+/+ siblings in this figure is needed to ensure that there isn't a background effect in the double.

- We repeated our skeletal stain of *ncl*$^{-/-}$*;p53*$^{-/-}$ mutants so that we could stain clutch mates with the *ncl*$^{-/-}$*;p53*$^{+/+}$ genotype. In our new experiment, performed with two different clutches and over 25 embryos identified for each genotype reported, we observe worsening of the craniofacial phenotype of *ncl*$^{-/-}$*;p53*$^{-/-}$ embryos. Prior staining experiment has been provided below to showcase the range of phenotypes we observe in *ncl*$^{-/-}$*;p53*$^{-/-}$ embryos. The neurocranium is barely stained with alcian blue to measure trabeculae lengths, however is well stained with alizarin red. We do observe an increased head size and an improvement in the shape of the meckel's cartilage in *ncl*$^{-/-}$*;p53*$^{-/-}$ compared to *ncl*$^{-/-}$*;p53*$^{+/+}$. We do not think that the light stain is an artifact, because we have performed this staining in five different clutches with over 80 *ncl*$^{-/-}$*;p53*$^{-/-}$ embryos identified and observe the

© 2022. Published by The Company of Biologists under the terms of the Creative Commons Attribution License (https://creativecommons.org/licenses/by/4.0/).

lighter staining only in this particular genotype. We would like to be cautious and not report this as a rescue.

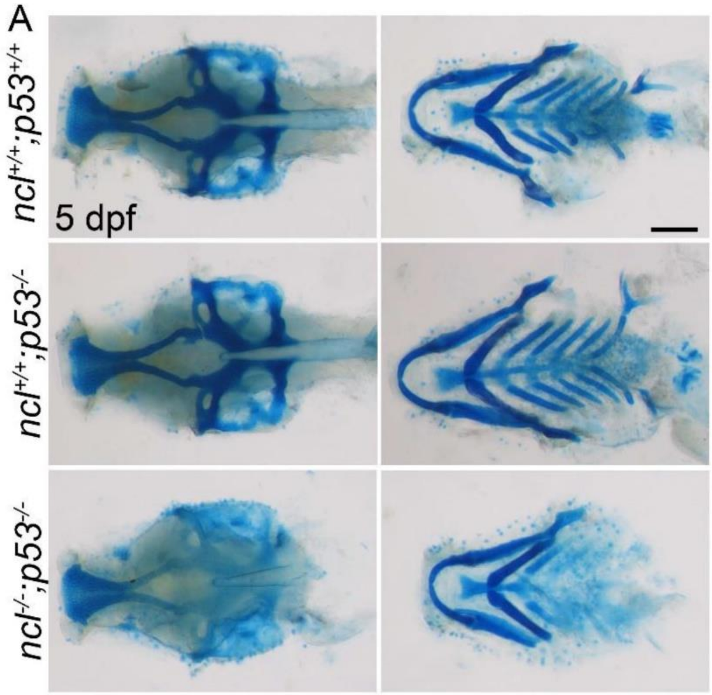


4). In a related concern, the morpholino data are highly concerning. The p53 morpholino-injected ncl mutants are much more disrupted than the double mutants. Even the control morpholino-injected mutants appear much worse than the ncl single mutants in Fig. 2J. Perhaps the ncl mutants are merely more sensitive to off target morpholino defects?

- We agree with the reviewer that the morpholino effect of p53 could be due to off target effects as well as possibly due to microinjection techniques. Therefore, we removed it from the paper as per Reviewer 1's suggestion.

5) In general, the craniofacial phenotypes are not quantified. This makes it very difficult to ascertain the overall level of rescue/interaction in their various experiments. The authors should add quantification of these effects. Even simple measures like percentage of embryos with a basihyal and average number of ceratobranchials/teeth would be very helpful.

- We have now added number of embryos where we observe rescue as compared to number of embryos observed with the particular genotype in parenthesis near the phenotype rescued throughout the result section.

Minor concerns:

1). In the legend for Fig. 1 the authors state "Nucleolin expression is specific to the jaw of the embryo." Some different wording should be used because it does appear to be expressed elsewhere. Even within the head it is not limited to the jaw.

- We have now changed the description of Nucleolin expression to "In 3 dpf wildtype zebrafish, Nucleolin expression is highly expressed in the jaw of the embryo."

2). In the legend for Fig. 3 panel E is not referenced. There appears to be a typo, labeling it as D.

- We thank the reviewer for pointing out our typographical error. This has been corrected.

3). In Fig. 3, it is not clear that apoptosis is restricted to the MHB. Separating the red and blue channels would help the reader assess this claim.

- We separated the red and blue channels for better visualization.

4). Line 292, "oth" should be "other".

- Thank you for pointing out our typographical error. This has been corrected.

© 2022. Published by The Company of Biologists under the terms of the Creative Commons Attribution License (https://creativecommons.org/licenses/by/4.0/).

5) In the methods the authors state they used the Walker and Kimmel bone and cartilage labeling protocol but then reference Kimmel 1995. Neither reference is in the reference list.

- Thanks for alerting us to this omission. We added Walker and Kimmel, 2007 to our reference list.

6) In many figures it is difficult to tell where there are significant differences between groups. A supplemental table listing the p values for all comparisons would be helpful.

- Great idea. We have added a supplementary table 1 with p-value comparisons as suggested

---

Second decision letter

MS ID#: DEVELOP/2021/200349

MS TITLE: Nucleolin loss-of-function leads to aberrant FGF signaling and craniofacial anomalies

AUTHORS: Soma Dash and Paul Trainor

I have now received all the referees reports on the above manuscript, and have reached a decision. The referees' comments are appended below, or you can access them online: please go to BenchPress and click on the 'Manuscripts with Decisions' queue in the Author Area.

The overall evaluation is positive and we would like to publish a revised manuscript in Development, provided that the Reviewer 1 comments can be satisfactorily addressed. Please attend to all the comments in your revised manuscript and detail them in a point-by-point response. If you do not agree with any of their criticisms, please explain clearly why this is so.

Reviewer 1

*Advance summary and potential significance to field*

A recurring phenomenon in neural crest (NC) biology is that NC cells are disproportionately impaired by mutation of genes presumed to be of ubiquitous expression and function, e.g., those involved in rRNA transcription and ribosome biogenesis. Numerous hypotheses have been put forward to explain this curiosity and it has been of interest to the Trainor lab for some time. In this manuscript, Dash & Trainor add nucleolin to this list of ubiquitous genes that NC cells are hypersensitive to the loss of. Ncl is a phosphoprotein that is a major component of the nucleolus (but also expressed in the cytoplasm) and has documented roles in rRNA processing and mRNA/DNA binding. Using an existing zebrafish insertional mutant allele for ncl, the authors show a clear downregulation of rRNA transcription and increased levels of p53 protein/apoptosis in mutant embryos, as well mild craniofacial malformations and lethality. They are able to rescue most of these phenotypes by mutating p53 or by treating with recombinant FGF8 or BMP2.
The authors have made numerous substantive changes to their manuscript and figures in response to the reviewers' concerns. The authors' description of the mutant craniofacial phenotype is improved, and they have added sample numbers, stages, and statistics where appropriate. Overall, the revised study is stronger and more scientifically rigorous now.

*Comments for the author*

I have three remaining disagreements with the authors about staining fidelity, which in turn make me question their conclusions about the mechanisms behind the reduced osteogenesis and Fgf8 activity.
1. Runx2 antibody staining: I appreciate that the authors have included a no-primary control in their response as well as a citation for published expression data. However, I still do not trust this staining pattern in Fig. 4.
Lack of staining in a no-primary control is not definitive proof that the antibody is recognizing the protein it is supposed to. The published images do not closely resemble what is shown here.

© 2022. Published by The Company of Biologists under the terms of the Creative Commons Attribution License (https://creativecommons.org/licenses/by/4.0/). 

Further, the prominent staining in Fig. 4E is in the intermandibularis anterior, intermandibularis posterior, interhyoideus, and hyohyoideus muscles (see McGurk 2017 (Fig. 1) or Schilling 1997 (Fig. 2) for muscle diagrams) not in the parasphenoid bone or palatoquadrate. It may be worth re-testing the antibody at a later stage (>48 hpf) when strong and specific Runx2a/b expression is known to be present around the earliest forming bones, rather than at this early stage when expression is diffuse or absent.
2. Alkaline phosphatase staining: Alkaline phosphatase is not specific to osteoblasts. Based on their morphology, the cells that stain for alkaline phosphatase activity in Fig. 4G-H look like they are the gill arteries, not in the ceratobranchial skeleton. These arteries are nicely labeled by the fli1a:GFP transgene.
3. Fgf8 antibody staining: I remain skeptical that this staining reflects the true distribution of Fgf8 protein despite the authors' argument. The mouse staining in the cited paper is still very specific, not nearly ubiquitous as appears here.
Additional comments:
Fig. 3I-J: Please note on the graphs what protein is being measured; i.e. p53 and p53/Mdm2.
Bmp2 is not incorporated into the final model. As both I and the reviewers noted, it is not currently possibly to verify that the Fgf8 rescue of craniofacial development works via Bmp2, but it is interesting that Fgf8 but not Bmp2 rescued rRNA transcription. Given that that BMH21 treatment did not preclude rescue of nlc mutants with Fgf8, the Bmp2 result really cements the interpretation that the growth factor-mediated skeletal rescue is not dependent on rRNA transcriptional rescue. This is briefly touched on in the second to last paragraph of the discussion, but I think it deserves further notice, at least in the model, because it is one of the most interesting results of the paper.
Citations for for fgf8a expression in the oral ectoderm:
Eberhart et al., Early Hedgehog signaling from neural to oral epithelium organizes anterior craniofacial development. 2006. Development 133: 1069-1077 Stock et al., Developmental genetic mechanisms of evolutionary toothloss in cypriniform fishes. 2006.
Development 133 :3127-3137.

Reviewer 2

*Advance summary and potential significance to field*

The authors show that nucleolin, a basic protein for nucleolar function, leads to decreased ribosomal synthesis and cartilage defects, through at least in part the stabilisation of fgf8a mRNA.

*Comments for the author*

All my concerns were addressed

Reviewer 3

*Advance summary and potential significance to field*

In their manuscript "Nucleolin loss-of-function leads to aberrant FGF signaling and craniofacial anomalies"
Dash and Trainor characterized ncl mutant zebrafish. They first characterize the expression of ncl demonstrating that it is maternally provided and expressed fairly ubiquitously, although enriched in some tissues such as the pharyngeal arches. They show that the craniofacial skeleton is malformed in ncl mutants. In ncl mutants, protein levels of p53 are transiently elevated and there is reduced immunoprecipitation of p53 with Mdm2. The elevation in p53 results in elevated apoptosis but only some phenotypes are rescued in ncl;p53 double mutants. They find that migration into the pharyngeal arches appears normal in ncl mutants, but that chondrogenesis and osteogenesis are disrupted, with Sox9a being downregulated and Runx2 being upregulated. This finding lead to the Fgf pathway as a potential mechanism. They show that Ncl binds fgf8a mRNA and, in the absence of ncl, the levels of fgf8a mRNA and protein are reduced. The addition of exogenous Fgf8 partially restores craniofacial structures and elevates the levels of rRNA transcripts. The rescue occurs even when Pol I is chemically inhibited suggesting that the mechanism is independent of the role of Ncl in rRNA biogenesis. They go on to demonstrate that Fgf8 stimulation elevates the levels of bmp2

© 2022. Published by The Company of Biologists under the terms of the Creative Commons Attribution License (https://creativecommons.org/licenses/by/4.0/). 

and that Bmp treatment can rescue ncl mutant embryos, suggesting this as the mechanism. The demonstration of non-rRNA functions for Ncl will be of broad interest to developmental biologists and, thus, warrants publication in Development.

*Comments for the author*

I have no remaining concerns regarding the manuscript.

---

**Second revision**

Author response to reviewers' comments

Reviewer 1 Comments for the Author:
I have three remaining disagreements with the authors about staining fidelity, which in turn make me question their conclusions about the mechanisms behind the reduced osteogenesis and Fgf8 activity.
1. Runx2 antibody staining: I appreciate that the authors have included a no-primary control in their response as well as a citation for published expression data. However, I still do not trust this staining pattern in Fig. 4. Lack of staining in a no-primary control is not definitive proof that the antibody is recognizing the protein it is supposed to. The published images do not closely resemble what is shown here. Further, the prominent staining in Fig. 4E is in the intermandibularis anterior, intermandibularis posterior, interhyoideus, and hyohyoideus muscles (see McGurk 2017 (Fig. 1) or Schilling 1997 (Fig. 2) for muscle diagrams) not in the parasphenoid bone or palatoquadrate. It may be worth re-testing the antibody at a later stage (>48 hpf) when strong and specific Runx2a/b expression is known to be present around the earliest forming bones, rather than at this early stage when expression is diffuse or absent.

- We agree that our Runx2 expression pattern is similar to the craniofacial musculature, however, the pattern of Runx2 immunostaining is similar to the expression pattern of *runx2a* and *runx2b* in-situ hybridization that have been previously published in the literature (Li et al., 2009). While we understand that the antibody has not been previously used for zebrafish immunostaining experiments, we and others have successfully used it for recognizing Runx2 in mice (Dash et al., 2020, JDR). The antibody is designed against human RUNX2 at 300-400 amino acid, which is a conserved region in humans, mice and zebrafish (85%). This information is now added to the paper.
- The reviewer suggested staining embryos after 48hpf due to concerns with the labelling pattern at 36hpf. We have indeed performed Runx2 immunostaining at 72 hpf, a stage where osteoblast differentiation has definitively occurred in concert with a very restricted pattern of Runx2 expression in the craniofacial region. While we could perform Runx2 immunostaining at 48-52 hpf, we think the staining at 72 hpf supersedes the need for staining at 48-52 hpf. To avoid any confusion and concerns with non-osteoblast Runx2 staining at 36 hpf, we have removed this data from the manuscript and replaced it with a *runx2a* in-situ hybridization, which shows upregulated runx2a expression in the embryo. The expression pattern of *runx2a* by in-situ hybridization constitutes a subset of Runx2 immunostaining due to the fact that Runx2 immunostaining labels both Runx2a and Runx2b.

2. Alkaline phosphatase staining: Alkaline phosphatase is not specific to osteoblasts. Based on their morphology, the cells that stain for alkaline phosphatase activity in Fig. 4G-H look like they are the gill arteries, not in the ceratobranchial skeleton. These arteries are nicely labeled by the fli1a:GFP transgene.

- We agree that endogenous alkaline phosphatase is expressed in cells other than the osteoblasts and most notably in arteries. However, we modified our staining protocol from Kamei et al.,2010, Methods Cell Biol to obtain staining in the osteoblasts. Further, if we overlay our skeletal preparation (Fig. 2O) with that of the alkaline phosphatase staining (Fig. 4H), the purple stain of alkaline phosphatase staining overlies the alcian blue staining. We agree that gill arteries are affected in the mutant too, which we will explore in the future.

© 2022. Published by The Company of Biologists under the terms of the Creative Commons Attribution License (https://creativecommons.org/licenses/by/4.0/). 

- The reviewer pointed out that fli1a:GFP expression pattern is similar to our alkaline phosphatase staining. We agree with that statement considering fli1a:GFP is expressed in the neural crest cells along with the vasculature (Askary, et al., 2017, Development; Kwak et al, 2013, Mol Cells) and therefore coincides well with the alkaline phosphatase staining. This reference and accompanying clarification have been added to the manuscript.

3. Fgf8 antibody staining: I remain skeptical that this staining reflects the true distribution of Fgf8 protein despite the authors' argument. The mouse staining in the cited paper is still very specific, not nearly ubiquitous as appears here.

- The antibody is designed specifically against zebrafish Fgf8a protein. Furthermore, it's important to note that since Fgf8a is a secreted protein, there will be differences in the mRNA and protein expression patterns.
- Nonetheless, we have performed in-situ hybridization to observe spatial expression of *fgf8a* mRNA (Fig. S6) and observe a downregulation of the mRNA similar to our qPCR experiments (Fig. 5B), which we have added to our supplementary material.

Additional comments:
Fig. 3I-J: Please note on the graphs what protein is being measured; i.e. p53 and p53/Mdm2.

- We have added the additional information to our graphs.

Bmp2 is not incorporated into the final model. As both I and the reviewers noted, it is not currently possibly to verify that the Fgf8 rescue of craniofacial development works via Bmp2, but it is interesting that Fgf8 but not Bmp2 rescued rRNA transcription. Given that that BMH21 treatment did not preclude rescue of *nlc* mutants with Fgf8, the Bmp2 result really cements the interpretation that the growth factor-mediated skeletal rescue is not dependent on rRNA transcriptional rescue. This is briefly touched on in the second to last paragraph of the discussion, but I think it deserves further notice, at least in the model, because it is one of the most interesting results of the paper.

- We appreciate this comment and have added Bmp2 to our model in Fig. 7 as requested.

Citations for fgf8a expression in the oral ectoderm:

Eberhart et al., Early Hedgehog signaling from neural to oral epithelium organizes anterior craniofacial development. 2006. Development 133: 1069-1077

Stock et al., Developmental genetic mechanisms of evolutionary toothloss in cypriniform fishes. 2006. Development 133 :3127-3137.

- We have now added these to our reference list and oral ectoderm to the regions of *fgf8a* expression.

---

<u>Third decision letter</u>

MS ID#: DEVELOP/2021/200349

MS TITLE: Nucleolin loss-of-function leads to aberrant FGF signaling and craniofacial anomalies

AUTHORS: Soma Dash and Paul Trainor
ARTICLE TYPE: Research Article

I am satisfied with the response to review and the revision of the manuscript. Your paper has been accepted for publication in Development, pending our standard ethics checks.

© 2022. Published by The Company of Biologists under the terms of the Creative Commons Attribution License (https://creativecommons.org/licenses/by/4.0/).
